# A photochemical C=C cleavage process: toward access to backbone *N*-formyl peptides

**DOI:** 10.3762/bjoc.17.202

**Published:** 2021-12-15

**Authors:** Haopei Wang, Zachary T Ball

**Affiliations:** 1 Department of Chemistry, Rice University, Houston TX, USA

**Keywords:** formyl peptide, nitroaryl compound, nitroso compound, olefin cleavage, photocleavage

## Abstract

Photo-responsive modifications and photo-uncaging concepts are useful for spatiotemporal control of peptides structure and function. While side chain photo-responsive modifications are relatively common, access to photo-responsive modifications of backbone N–H bonds is quite limited. This letter describes a new photocleavage pathway, affording *N*-formyl amides from vinylogous nitroaryl precursors under physiologically relevant conditions via a formal oxidative C=C cleavage. The *N*-formyl amide products have unique properties and reactivity, but are difficult or impossible to access by traditional synthetic approaches.

## Findings

The photochemistry of nitroaromatic functional groups has a rich history that dates back decades [1–5]. Photochemical pathways allow access to diverse and interesting target structures [6–10], though photocleavage of C–X bonds for use as photoremovable protecting groups [11–12] has been the major thrust of the development of 2-nitroaryl compounds. Various 2-nitrobenzyl derivatives are used to photocage heteroatom functional groups, including alcohols, amines, carboxylic acids, and phosphates [11]. Typical photochemical pathways result in cleavage of a benzylic C–X bond following initial benzylic H-atom abstraction [11,13]. In contrast, photorelease systems based on C–C or C=C bond photocleavage are quite rare [14–15]. We recently reported a vinylogous analogue of this photo-deprotection process, which allowed photocleavage of alkenyl sp^2^ C–X bonds, rather than benzylic sp^3^ C–X cleavage [16–17]. We now report that further studies into this reaction demonstrate two mechanistically distinct photocleavage pathways, with selectivity dependent on pH. In addition to an anticipated alkenyl sp^2^ C–X bond cleavage pathway, we identified a new photochemical reaction pathway, prevalent under neutral and acidic reaction conditions, which leads to formyl products from formal oxidative cleavage of a C=C bond.

**Figure 1:**
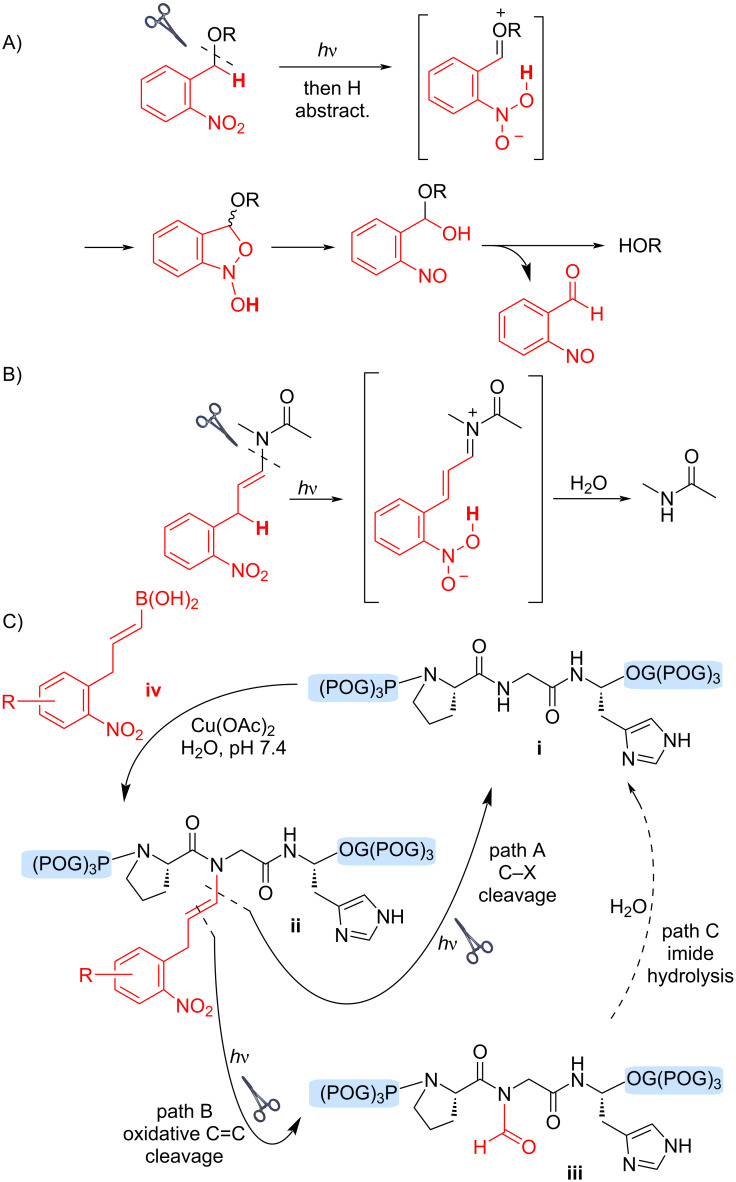
Uncaging of peptide backbone N–H bonds from Chan–Lam-type modification.

Our interest in vinylogous analogues of 2-nitroaryl photoreactive groups stems from studies into alkenylboronic acid reagents for Chan–Lam-type modification of peptide backbone N–H bonds, directed by a proximal histidine residue (Figure 1C, step, **i** + **iv** → **ii**) [18–20]. Subsequent investigations validated the use of photoreactive boronic acids as an approach to reversible backbone N–H modification via photocleavage of an alkenyl C–N bond [16–17]. Traditional 2-nitroaryl groups allow cleavage of benzylic C–X bonds (e.g. C–O cleavage, Figure 1A) through H-atom abstraction from a photoexcited intermediate, which produces an oxonium-type intermediate (in brackets). Hydrolysis of this intermediate then affords an alcohol product. Recently [16–17], we demonstrated that vinylogous analogues of this mechanism (Figure 1B) provide entry into similar photo-uncaging chemistries for amide release.

Figure 1C shows an example of this concept applied to a peptide substrate. Reaction of the peptide **i** with an alkenylboronic acid reagent **iv** in the presence of a copper(II) salt in water provides access to the backbone N–H alkenylation product **ii**, directed by a neighboring histidine residue. Upon exposure to 365-nm light, photocleavage of the caging group (red) was observed, producing the free peptide **i**. Irradiation longer than 10 minutes were sometimes necessary for maximal yield of photo-deprotected product **i** [16]. Furthermore, byproducts or transiently stable intermediates were sometimes indicated by HPLC and/or NMR of these photocleavage reactions [16–17]. These observations prompted a more detailed study of the components present during photocleavage reactions of small-molecule models, leading to the identification of the *N*-formyl product **iii**, a possible intermediate on the path to product **i** via imide hydrolysis.

**Figure 2:**
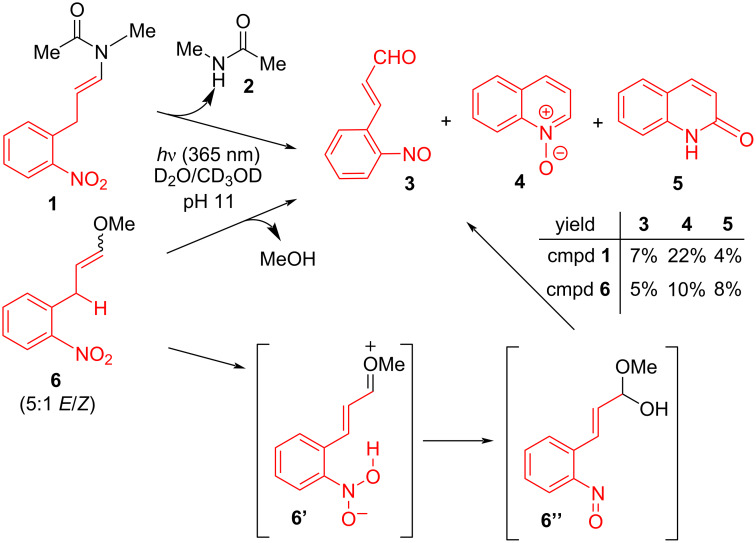
Photocleavage of compounds **1** and **6** under basic conditions. Yield of products was calculated from crude ^1^H NMR using residual CD*_3_*OD peaks as internal standard.

To better understand the mechanism of photocleavage and the appearance of the formyl product **iii**, we first identified the 2-nitroaryl-derived byproducts produced in this reaction. Model compound **1** was subjected to aqueous photocleavage in the presence of triethylamine, and the resulting reaction mixture was purified by reversed-phase HPLC (Figure 2). We isolated a nitroso product **3**, in addition to two other major identifiable components of the crude reaction: quinoline *N*-oxide (**4**) and quinolinone (**5**). The compounds **4** and **5** are C_9_ compounds possibly derived from thermal or photochemical rearrangement of compound **3** or another intermediate. The yield of each product was calculated by NMR and verified by isolation (Figure 2). To test the generality of this process with other functional groups, we prepared and tested alkenyl ether **6** as a model of C–O-bond cleavage. Photoirradiation of the ether **6** similarly provided a mixture of C_9_**-**containing products **3**, **4**, and **5**. Under these reaction conditions, the C–X cleavage products (MeOH or **2**) were observed, but no formyl products were formed. The C_9_ byproducts – the nitroso **3**, and related compounds **4** and **5** are all consistent with the classical C–X cleavage mechanism and with hydrolysis of the presumed oxonium intermediate **6’**, but are inconsistent with the production of formyl products.

**Figure 3:**
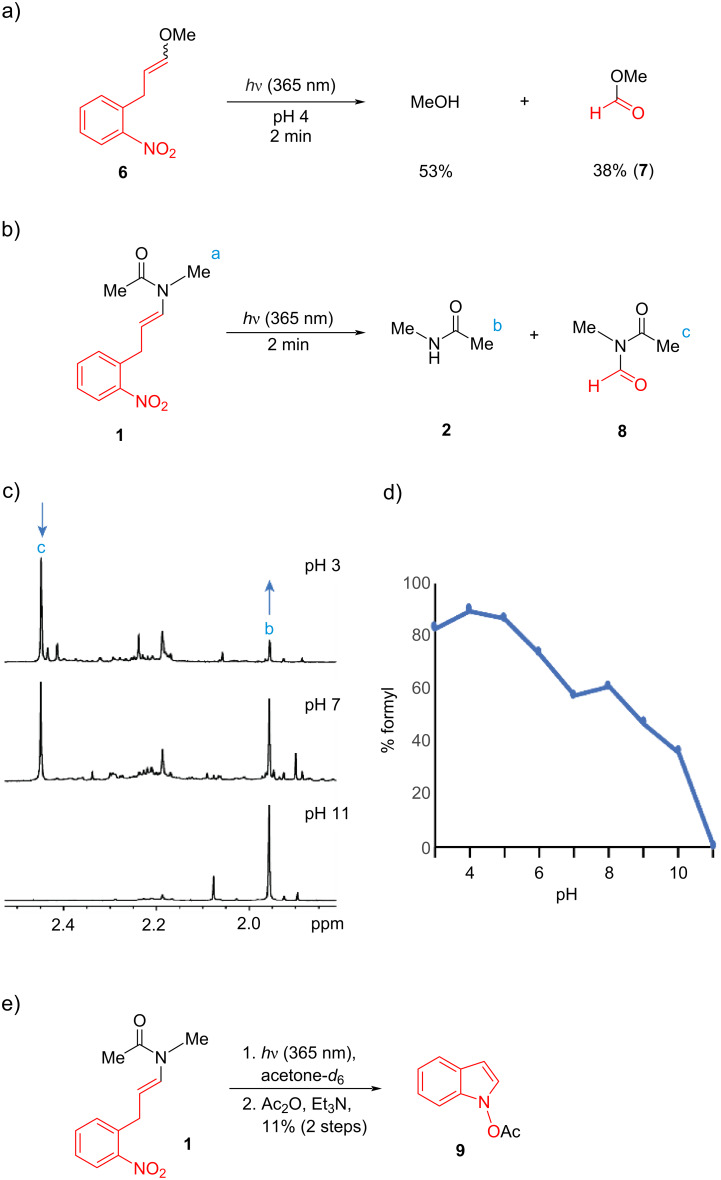
(a) Photocleavage of compound **6** under acidic conditions. Yields determined by ^1^H NMR using residual CD_3_OD as an internal standard. (b–d) Selectivity of photocleavage of alkenyl amide **1** as a function of pH. Product percentage of *N*-formyl **8** was assessed by crude NMR (c) and graphed (d). Formation of *N*-formyl-*N*-methyl acetamide **8** during photocleavage of compound **1**. Conditions: **1** (1.8 μmol) was dissolved in MeOD-*d*_4_ (200 μL) and deuterated buffer (400 μL). The solution was irradiated at 365 nm for 2 min. (e) Photocleavage reaction of **1** in acetone.

In contrast, photoirradiation of the same alkenyl ether **6** under acidic conditions at pH 4.0 provided a mixture of methanol (53%) and methyl formate (38%, **7**) as determined by NMR, the latter product is the result of formal oxidative C=C cleavage (Figure 3a). Alkenyl amide **1** at pH 4.0 similarly gave mixtures of the C–N cleavage product **2** and C=C cleavage product **8**. We examined product selectivity in the irradiation of alkenyl amide **1** across a range of pH and found a significant correlation (Figure 3b–d). The formyl product **8** predominated at acidic and neutral pH. The amount of **8** decreased with increasing pH, and above pH 10 the C–X cleavage product **2** became the major product. Unfortunately, no products other than the formyl compound were isolated after photocleavage of compound **1** or **6** in acidic conditions. Instead, when irradiation of alkenyl amide **1** was conducted in acetone, crude NMR analysis indicated the appearance of product **8** as well as new peaks in the aromatic region.

**Figure 4:**
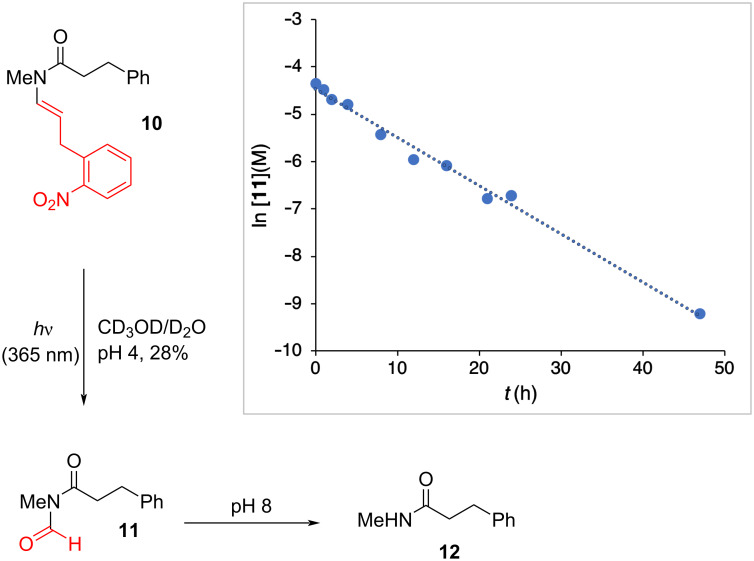
Preparation and hydrolysis kinetics (inset) of *N*-formyl product **11**. Dashed line: first-order decay fit used in calculating the rate constant.

Following acetylation of the reaction mixture, we were able to isolate small quantities of *O*-acetyl *N-*hydroxyindole (**9**, Figure 3e), although the initial byproduct *N*-hydroxyindole itself proved too unstable to be isolated. It is noteworthy within this context that hydroxyindole is a C_8_ compound, consistent with transfer of the C_1_ formyl group to compound **8**. The formation of amide **2** at elevated pH could, in theory, derive from hydrolysis of the initially formed formyl product **8** (i.e. Figure 1C). However, the appearance of primarily C_9_ byproducts in the formation of amide **2** at elevated pH precludes pathways involving the intermediacy of **8**. To provide additional support for this analysis, and to assess the stability of *N*-formyl amides formed in this reaction, we irradiated alkenyl amide **10**, which contains a 2-phenylethyl substituent that allowed easier isolation of *N*-formyl **11** (Figure 4). After irradiation, the product **11** was isolated in 28% yield, the modest yield reflecting the instability in water and on silica of this compound. The purified *N*-formyl **11** was then dissolved in buffer (pH 8), and its hydrolysis to amide **12** was assessed (Figure 4, inset). We observed clean first-order kinetics to give amide **12** with a half-life (*t*_1/2_) of 6.4 h.

**Figure 5:**
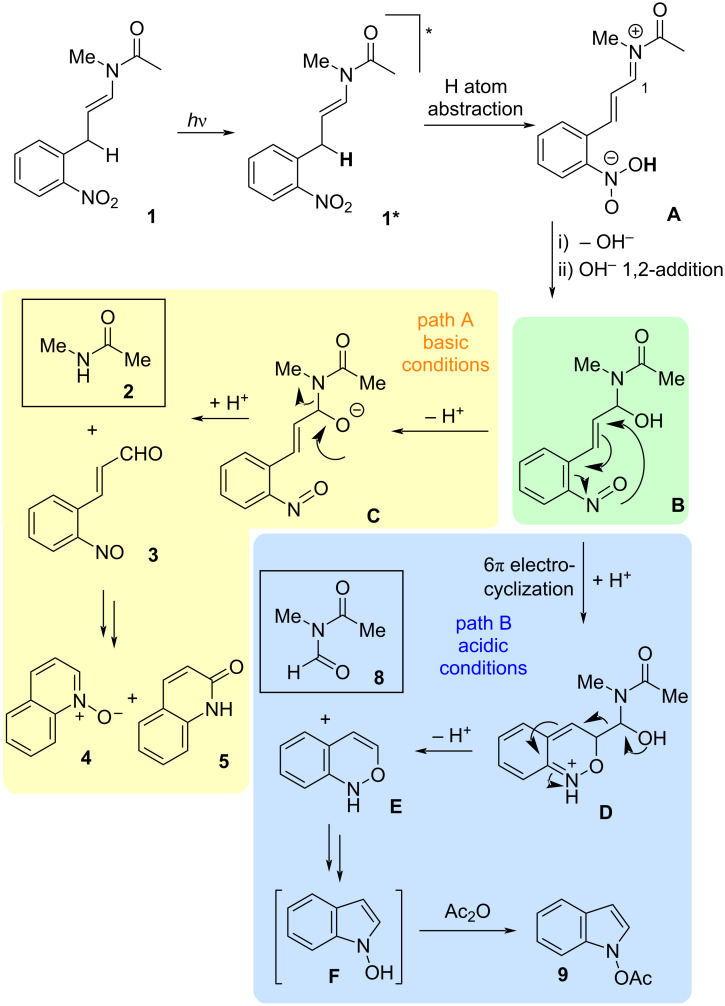
Proposed mechanism for the formation of aldehyde **3** and *N*-formyl product **8**.

The observation of *N*-formyl products can be rationalized with a bifurcating mechanism (Figure 5). Following photoactivation, H-atom abstraction and nucleophilic addition of water would produce the key intermediate **B**. Such hemi-aminal compounds would be unstable under basic conditions, readily forming aldehyde products **3**. However, related hemi-aminal compounds are quite stable under non-basic conditions, and the motif is even contained in some natural products, such as zampanolide [21] and spergualin [22]. We propose a competing electrocyclization pathway, affording the heterocycle **D**, a pathway which should not be base-catalyzed, and thus may be reasonably predominant under appropriate conditions. From heterocycle **D**, a C–C cleavage would produce the *N*-formyl product **8** and a re-aromatized C_8_ heterocyclic byproduct **E**. Rearrangement to hydroxyindole (**F**) would then account for the isolation of the acetylated analogue **9**.

The photochemical pathway described here represents a formal oxidative olefin cleavage of vinylogous nitroaryl-modified amides and ethers. The pathway adds to the diversity of photochemical pathways known for 2-nitrophenyl systems, and the concept described here might be useful for the synthetic unmasking of relatively sensitive imido structures. For chemical biology applications, the results point to a far more diverse photochemistry than previously assumed for vinylogous photocleavage systems. Although formyl hydrolysis to the “expected” amide products can and does occur under physiological conditions, the rates of this hydrolysis are slow for the simple models in this study. Within more complex peptides or proteins, selectivity in photocleavage pathways may differ significantly, depending on local chemical environment. It is also worth noting that *N*-formyl products are themselves acylating reagents, and thus could find use in photochemical generation of selective acyl donors.

## Supporting Information

File 1 Experimental section and additional information.
