# Supplementary material for: A photochemical C=C cleavage process: toward access to backbone N-formyl peptides
Source: Beilstein J Org Chem. 2021 Dec 15;17:2932–8. doi: 10.3762/bjoc.17.202 (PMC8685554; doi:10.3762/bjoc.17.202)
Supplement: File 1 — Experimental section and additional information. [file Beilstein_J_Org_Chem-17-2932-s001.pdf]

BEILSTEIN JOURNAL OF ORGANIC CHEMISTRY

Supporting Information

for

A photochemical C=C cleavage process: toward access to backbone *N*-formyl peptides

Haopei Wang and Zachary T. Ball

Beilstein J. Org. Chem. **2021,** *17,* 2932–2938. doi:10.3762/bjoc.17.202

Experimental section and additional information

License and Terms: This is a supporting information file under the terms of the Creative Commons Attribution License (https://creativecommons.org/licenses/by/4.0). Please note that the reuse, redistribution and reproduction in particular requires that the author(s) and source are credited and that individual graphics may be subject to special legal provisions.
The license is subject to the *Beilstein Journal of Organic Chemistry* terms and conditions: (https://www.beilstein-journals.org/bjoc/terms)

Table of Contents

General information and methods 2
Experimental procedures 3
Irradiation experiments 3
Preparation of reagents 5
Supplementary data 8
For figure 2 8
For figure 3 13
For figure 4 16
Compound Characterization 19
HPLC of synthesized compounds 19
ESI-MS of synthesized compounds 19
^{1}H and ^{13}C NMR spectra of purified compounds 21

General information and methods

Chemicals

All chemicals used were purchased from commercial suppliers and used without further purification. 3-Phenylpropionyl chloride (**S1**) was purchased from Oakwood Chemicals (cat. #448612). *p*-Toluenesulfonic acid monohydrate was purchased from Sigma-Aldrich (cat. #402885). *N*,*N*-Diformylacetamide was purchased from Acros Organics (cat.# 380450010). (Methoxymethyl)triphenylphosphonium chloride was purchased from MilliPore Sigma (cat.# 309567). Triethylamine hydrochloride ($Et_3N{\cdot}HCl$) was purchased from Fluka (cat.#90350). Acetic acid-d_4 was purchased from Sigma-Aldrich (cat. #416886). Sodium acetate-d_3 was purchased from Sigma Aldrich (cat.#176079). Deuterium oxide (D_2O) was purchased from Cambridge Isotope Laboratories, Inc. Methyl formate was purchased from Oakwood Chemicals (cat. #101914).

Buffer

Et_3N/HCl buffer (10 mM, pH 10–11) was prepared by addition of Et_3N (3.48 μL, 0.025 mmol) and $Et_3N{\cdot}HCl$ (3.44 mg, 0.025 mmol) to D_2O (5 mL). The pH was adjusted by addition of aq HCl for pH 10 buffer.

Acetate buffer (10 mM, pH 3) was prepared by addition of acetic acid-d_4 (2.86 μL, 0.05 mmol) to D_2O (5 mL). The pH was adjusted by addition of aq NaOH or HCl.

Acetate buffer (10 mM, pH 4) was prepared by addition of sodium acetate-d_3 (0.98 mg, 0.0115 mmol) and acetic acid-d_4 (2.2 μL, 0.0385 mmol) to D_2O (5 mL). The pH was adjusted by addition of aq NaOH or HCl.

Acetate buffer (10 mM, pH 5) was prepared by addition of sodium acetate-d_3 (2.85 mg, 0.0335 mmol) and acetic acid-d_4 (0.94 μL, 0.0165 mmol) to D_2O (5 mL). The pH was adjusted by addition of aq NaOH or HCl.

Triethylammonium acetate buffer (10 mM, pH 6-9) was prepared by addition of Et_3N (6.97 μL, 0.05 mmol) and acetic acid-d_4 (2.86 μL, 0.05 mmol) to D_2O (5 mL). The pH was adjusted by addition of aq NaOH or HCl.

Synthesis of known compounds:

(*E*)-*N*-methyl-*N*-(3-(2-nitrophenyl)prop-1-en-1-yl)acetamide (**1**) was prepared according to a previously reported protocol.[1]
3-(2-nitrophenyl)propanal (**S2**) was prepare according to a previously reported protocol.[2]
From *N*,*N*-diformylacetamide was synthesized *N*-formyl-*N*-methyl-3-phenylpropanamide (**8**) as a product standard via a previously reported protocol.[3]
2-(2-nitrophenyl)acetaldehyde (**S3**) was prepared according to a previously reported protocol.[4]

Instrumentation

ESI-MS was conducted on a Bruker Daltonics MicroTOF spectrometer.

^{1}H and ^{13}C NMR spectra were obtained on Bruker AVANCE 600 spectrometer.

Reverse-phase HPLC (RP-HPLC) was performed on a Shimadzu CBM-20a instrument with Phenomenex Jupiter 4µ Proteo 90A (250 mm × 4.6 mm for analytical scale) and Phenomenex Jupiter 4µ Proteo 90A (250 × 15 mm for preparative scale) column. The flow rate was 1 mL/min for analytical scale and 8 mL/min for preparative scale. A gradient of acetonitrile/water with 0.1% trifluoroacetic acid (TFA) was employed. Compounds were detected by UV detector at 220 nm and 280 nm.

Photoreactor used in the photocleavage reactions was a Penn PhD Photoreactor m2 with a UV LED light lamp (365 nm). The LED light source was set to 50% intensity.

Experimental procedures

Irradiation experiments

Irradiation of nitroarene 1 under basic conditions

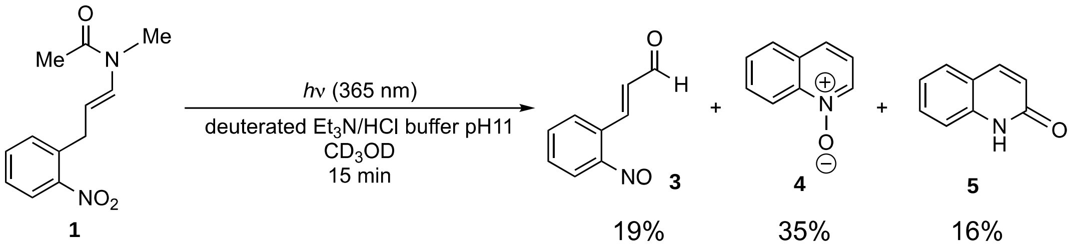

Compound **1** (10 mg, 0.043 mmol) was dissolved in CD_3OD (4.78 mL) and deuterated Et_3N/HCl buffer (10 mM, pH 11, 9.56 mL) in a 20-mL scintillation vial. After measuring a zero-time point NMR, the vial was irradiated using the Penn PhD Photoreactor m2 with a 365-nm LED light source set to 50% intensity. After 15 minutes, reaction was complete by NMR. The products were purified by RP-HPLC (15-75% MeCN over 22 min) to afford quinoline *N*-oxide (2.2 mg, 35%), quinolin-2(1*H*)-one (1.0 mg, 16%) and (*E*)-3-(2-nitrosophenyl)acrylaldehyde (1.3 mg, 19%).

Data for 4: ^{1}H NMR (600 MHz, CD_3OD) δ 8.71 (d, 1H, J = 6.1 Hz), 8.66 (d, 1H, J = 8.9 Hz), 8.19 (d, 1H, J = 8.5 Hz), 8.12 (d, 1H, J = 8.3 Hz), 7.94 (ddd, 1H, J = 9.2, 7.2,1.2 Hz), 7.81 (ddd, 1H, J = 8.6, 7.1, 0.9), 7.57 (dd, 1H, J = 8.8, 6.0 Hz). Data is consistent with that previously reported.[5]

Data for 5: ^{1}H NMR (600 MHz, CD_3OD) δ 7.98 (d, 1H, *J* = 9.5 Hz), 7.68 (dd, 1H, *J* = 7.8, 1.0 Hz), 7.56 (ddd, 1H, *J* = 8.3, 7.2, 1.3 Hz), 7.37 (d, 1H, *J* = 8.3 Hz), 7.27 (ddd, 1H, *J* = 8.3, 7.1, 0.9 Hz), 6.62 (d, 1H, *J* = 9.5 Hz). Data is consistent with that previously reported.[6]

Data for 3: ^{1}H NMR (600 MHz, CD_3OD) δ 9.74 (d, 1H, *J* = 7.6 Hz), 8.11 (dd, 1H, *J* = 8.1, 0.8 Hz), 8.08 (d, 1H, *J* = 15.9 Hz), 7.88 (dd, 1H, *J* = 7.9, 1.0 Hz), 7.78 (td, 1H, *J* = 7.5, 0.7 Hz), 7.70-7.67 (m, 1H), 6.74 (dd, 1H, *J* = 15.9, 7.6 Hz). ^{13}C NMR (151 MHz, CD_3OD): δ 195.5, 149.3, 135.0, 133.6, 132.6, 130.5, 126.2, 125.5 (The **C**–NO was not observed). IR (thin film): 1683, 1607, 1523, 1442, 1348, 1204, 1142, 1125, 977, 864, 844, 802, 738, 724 cm^{-1}. HPLC t_R: 17.9 min (Ramp: 15-90% MeCN 22mins).

Irradiation of nitroarene 1 in acetone to give 10 and 12

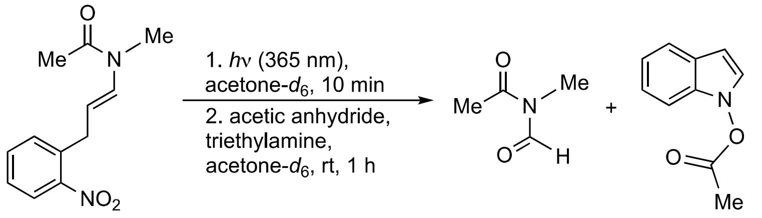

1 **10** **12**

Compound **1** (9.9 mg, 0.042 mmol) was dissolved in acetone-d_6 (14.2 mL) in a 20-mL scintillation vial. After measuring a zero-time point NMR, the vial was irradiated using the Penn PhD Photoreactor m2 with a 365-nm LED light source set to 50% intensity. After 10 minutes, reaction was complete by NMR. Acetic anhydride (16.0 μL, 0.17 mmol) and triethylamine (23.6 μL, 0.17 mmol) were added to the reaction. The reaction mixture was then stirred at room temperature for 1 h. One of the acetylated photocleavage products, 1H-indol-1-yl acetate, was purified by preparative TLC (ethyl acetate/hexanes, 1:4 with 0.1% triethylamine) as a red oil (0.8 mg, 11%). 1H-indol-1-yl acetate: ^{1}H NMR (600 MHz, acetone-d_6) δ 7.58 (d, 1H, *J* = 8.0 Hz), 7.37 (d, 1H, *J* = 3.6 Hz), 7.32 (dd, 1H, *J* = 8.2, 0.7 Hz), 7.20 (m, 1H), 7.09 (m, 1H), 6.47 (dd, 1H, *J* = 3.5, 0.7 Hz), 2.43 (s, 3H). ^{13}C NMR (151 MHz, acetone-d_6): δ 169.5, 134.6, 126.3, 125.6, 123.5, 121.9, 121.2, 109.2, 99.9, 18.1. ESI-MS: calcd for $C_{10}H_9NO_2$ $[M-Ac+2H]^+$ 134.1, found 134.1. ^{1}H NMR spectrum is consistent with a previous report.[7]

Irradiation of compound 6 under basic conditions

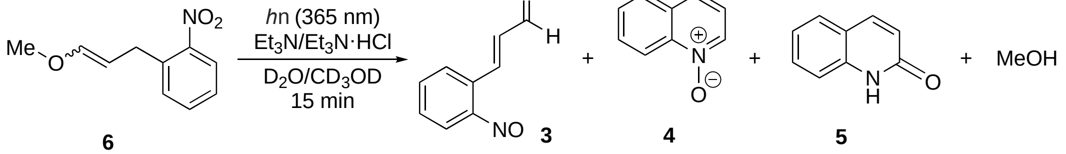

6 **3** **4** **5**

5% 8% 3%

Compound **6** (5.0 mg, 0.026 mmol) and $Et_3N\cdot HCl$ (4.2 mg, 0.031 mmol) were dissolved in CD_3OD (3.5 mL) in a 20-mL scintillation vial. Et_3N (3.45 μL, 0.025 mmol) and D_2O (3.75 mL) were added to the mixture. After measuring a zero-time point NMR, the vial was irradiated using the Penn PhD Photoreactor m2 with a 365-nm LED light source set to 50% intensity. After 15 minutes, reaction was complete by NMR. The products were purified by RP-HPLC (15-90%

MeCN over 22 min) to afford quinoline *N*-oxide (0.3 mg, 8%), quinolin-2(1*H*)-one (0.1 mg, 3%) and (*E*)-3-(2-nitrosophenyl)acrylaldehyde (0.2 mg, 5%). Methanol peak in the crude reaction mixture was confirmed by comparing with literature references.[8,9]

Irradiation of compound 6 under acidic conditions

Compound **6** (1.0 mg, 0.0052 mmol) was dissolved in methanol-d_4 (571 μL) and deuterated acetate buffer pH 4 (1.14 mL) in a 4-mL scintillation vial. After measuring a zero-time point NMR, a 600-μL aliquot was irradiated using the Penn PhD Photoreactor m2 with a 365-nm LED light source set to 50% intensity. After 2 minutes, reaction was complete by NMR.

Buffer pH screening for compound 1

Compound **1** (0.42 mg, 0.0018 mmol) was dissolved in CD_3OD (200 μL) and deuterated buffer (pH 3-11, 400 μL). After taking a zero-time point NMR, the samples were irradiated using the Penn PhD Photoreactor m2 with a 365-nm LED light source set to 50% intensity for 2 min. Two-minute time point NMR was taken for each sample.

Preparation of reagents

Preparation of 1-(3-methoxyallyl)-2-nitrobenzene (6)

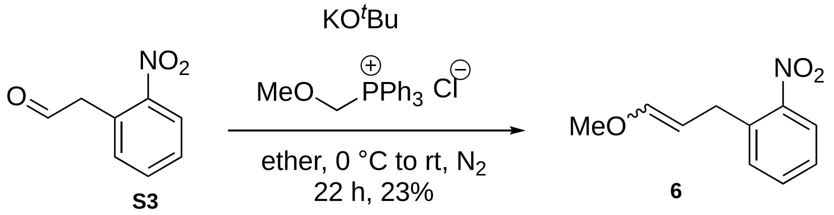

Adapting form a previous procedure,[10] to a round bottom flask was added (methoxymethyl)triphenylphosphonium chloride (3.1 g, 9.1 mmol). The solid was purged under N_2. Then dry ether (92 mL) was added to the flask. Potassium *t*-butoxide (883 mg, 7.9 mmol) was first dissolved in dry ether (40 mL) under nitrogen and added to the flask. The mixture was stirred at 0 °C for 30 min. Then the mixture was cooled to -70 °C, and a solution of **S3** (1.0 g, 6.1 mmol) in dry ether (66 mL) was added dropwise to the flask. The mixture was stirred at 0 °C for 30 min. Then the mixture was allowed to warm to rt and stirred for 20 h. The reaction was quenched by adding water, and then extracted with ethyl acetate (150 mL × 3). The organic layer was washed with brine (100 mL) and dried with Na_2SO_4. The solvent was removed under reduced pressure. The crude material was purified by flash chromatography (ethyl acetate/hexanes, 1:20) to afford a yellow oil **6** as a mixture of olefin isomers (268 mg, 23% yield, 5:1 trans/cis). ^{1}H NMR (600 MHz, $CDCl_3$) δ 7.88 (d, 0.8H, *J* = 8.2 Hz), 7.86 (d, 0.2H, *J* = 7.9 Hz), 7.53 (td, 0.9H, *J* = 7.5, 0.9 Hz), 7.50-7.49 (m, 0.2H), 7.45 (d, 0.2H, *J* = 7.8 Hz), 7.41 (d, 0.8H, *J* = 7.6 Hz), 7.36-7.33 (m, 0.9H), 7.32-7.30 (m, 0.2H), 6.45 (d, 0.8H, *J* = 12.6 Hz), 6.03 (d, 0.2H, *J* = 6.1 Hz), 4.87 (dt, 0.8H, *J* = 12.7, 7.4 Hz), 4.58 (dt, 0.2H, *J* = 6.2, 7.4 Hz), 3.67 (d, 0.4H, *J* = 7.4 Hz), 3.63 (s, 0.5H), 3.54 (d,

1.8H, *J* = 7.4 Hz), 3.53 (s, 2.6H). ^{13}C NMR (151 MHz, $CDCl_3$) δ 152.5, 151.0, 149.2, 148.0, 136.7, 136.5, 133.1, 133.0, 131.7, 131.5, 127.2, 126.9, 124.6, 124.4, 102.8, 99.6, 59.8, 56.0, 31.2, 27.4. ESI-MS: calcd for $C_{10}H_{11}NO_3$ $[M+H]^+$ 194.1, found 194.1.

Preparation of (*E*)-*N*-methyl-*N*-(3-(2-nitrophenyl)prop-1-en-1-yl)-3-phenylpropanamide (7)

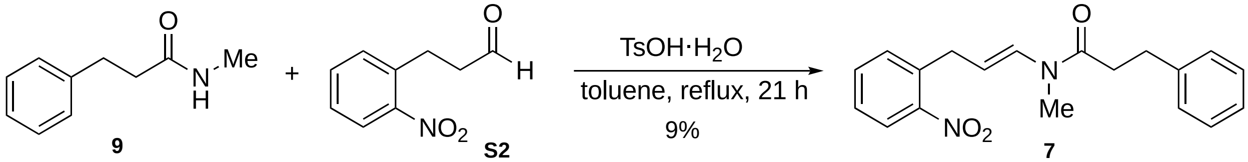

Adapting from a previous procedure,[1] to a 5-mL round bottom flask was added aldehyde **S2** (76 mg, 0.424 mmol), *p*-toluenesulfonic acid monohydrate (2.7 mg, 0.0142 mmol), amide **9** (208 mg, 1.27 mmol) and toluene (1.5 mL). The mixture was refluxed in a Dean–Stark apparatus for 21 h. The solvent was removed in reduced pressure, and the crude reaction mixture was purified by flash chromatography (ethyl acetate/hexanes, 1:4 with 0.1% triethylamine) to yield a pale yellow oil **7** as a 3:2 mixture of amide rotamers (12.4 mg, 9%). ^{1}H NMR (600 MHz, benzene-d_6) δ 7.77 (d, 0.4H, *J* = 14.5 Hz), 7.50-7.47 (m, 1H), 7.14-7.03 (m, 5H), 6.89-6.87 (m, 0.6H), 6.84-6.81 (m, 0.4H), 6.79-6.77 (m, 1H), 6.67-6.65 (m, 0.6H), 6.63-6.60 (m, 0.4H), 6.32 (d, 0.6H, *J* = 13.7 Hz), 4.88 (dt, 0.4H, *J* = 14.6, 7.2 Hz), 4.55 (dt, 0.6H, *J* = 13.7, 7.2 Hz), 3.36 (d, 0.8H, *J* = 7.1 Hz), 3.27 (d, 1.2H, *J* = 7.1 Hz), 2.98 (t, 1.2H, *J* = 7.7 Hz), 2.93 (t, 0.8H, *J* = 7.7 Hz), 2.75 (s, 1.8H), 2.36 (t, 1.2H, *J* = 7.8 Hz), 2.08-2.05 (m, 2H). ^{13}C NMR (151 MHz, benzene-d_6): δ 170.10, 170.08, 150.3, 149.9, 142.2, 142.1, 136.8, 136.1, 133.0, 132.8, 132.3, 131.5, 131.1, 130.1, 129.23, 129.21, 129.09, 129.06, 128.8, 127.6, 127.4, 126.7, 125.0, 124.8, 107.0, 106.3, 36.6, 36.2, 34.4, 33.9, 31.7, 31.6, 31.5, 29.6. ESI-MS: calcd for $C_{19}H_{20}N_2O_3$ $[M+H]^+$ 325.2, found 325.1.

Preparation of *N*-formyl-*N*-methyl-3-phenylpropanamide (8)

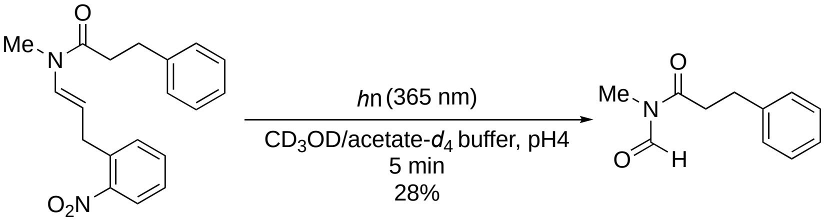

Compound **7** (6.3 mg, 0.019 mmol) was dissolved in CD_3OD (3.60 mL) and deuterated acetate buffer (pH 4, 1.8 mL). After measuring a zero-time point NMR, the vial was irradiated using the Penn PhD Photoreactor m2 with a 365-nm LED light source set to 50% intensity. After 5 min, reaction was complete by NMR. The solvent was removed by reduced pressure and lyophilization. The crude mixture was purified by flash chromatography (ethyl acetate/hexanes, 1:6 → 1:1) to afford *N*-formyl-*N*-methyl-3-phenylpropanamide (1.0 mg, 28%). ^{1}H NMR (600 MHz, $CDCl_3$) δ 9.22 (s, 1H), 7.33-7.30 (m, 2H), 7.25-7.21 (m, 3H), 3.11 (s, 3H), 3.07-3.04 (m, 2H), 2.95-2.92 (m, 2H). Characterization data is consistent with a previous report.[3]

Preparation of *N*-Methyl-3-phenylpropionamide (9)

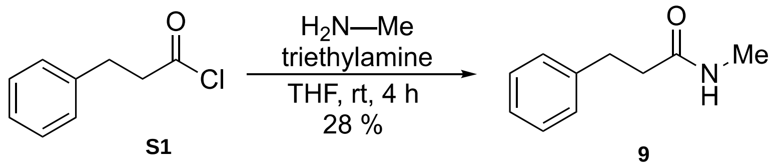

Adapting from a previous procedure,[11] to a round bottom flask was added tetrahydrofuran (5 mL) and 3-phenylpropionyl chloride (881 μL, 5.93 mmol). Then methylamine (40% in water, 6.1 mL, 71.2 mmol) and triethylamine (992 μL, 7.12 mmol) were added. The mixture was stirred at rt for 4 h. After 4 h, the reaction was acidified with HCl (4 M in dioxane), and then extracted with ethyl acetate (20 mL × 2). The organic phase was then washed with brine (10 mL × 2), dried with Na_2SO_4. The solvent was removed under reduced pressure. The crude material was recrystallized from ethyl acetate/hexane to afford white crystals (266 mg, 28%). ^{1}H NMR (600 MHz, $CDCl_3$) δ 7.29-7.27 (m, 2H), 7.21-7.19 (m, 3H), 5.47 (br s, 1H), 2.96 (t, 2H, J = 7.9 Hz), 2.77 (d, 3H, J = 4.5 Hz), 2.47 (t, 2H, J = 7.9 Hz). ESI-MS: calcd for $C_{10}H_{13}NO$ $[M+H]^+$ 164.1, found164.1. Data obtained is consistent with a previous report.[11]

Supplementary data

For figure 2

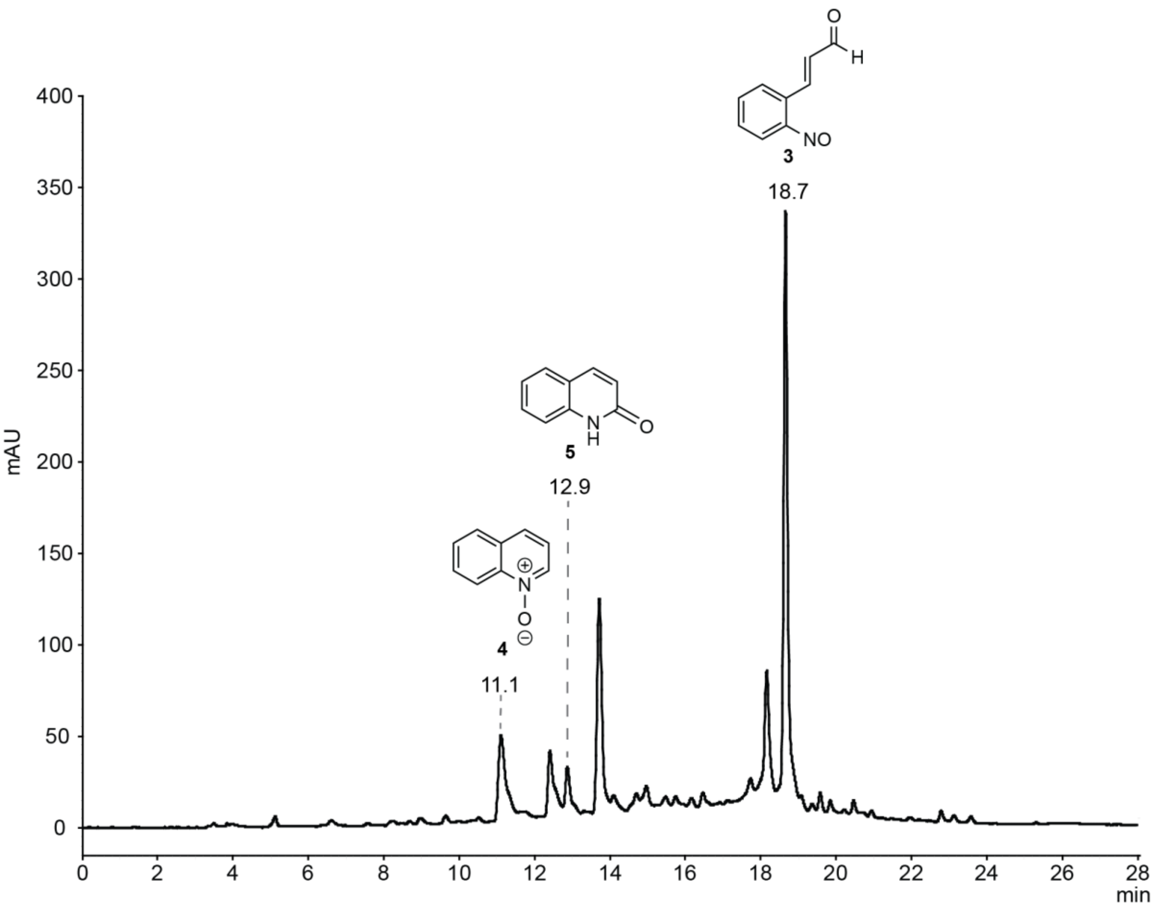

Figure S1. RP-HPLC trace of the crude photocleavage of compound **1**. Ramp: 15-75% MeCN 22 mins. Absorbance: 280 nm.

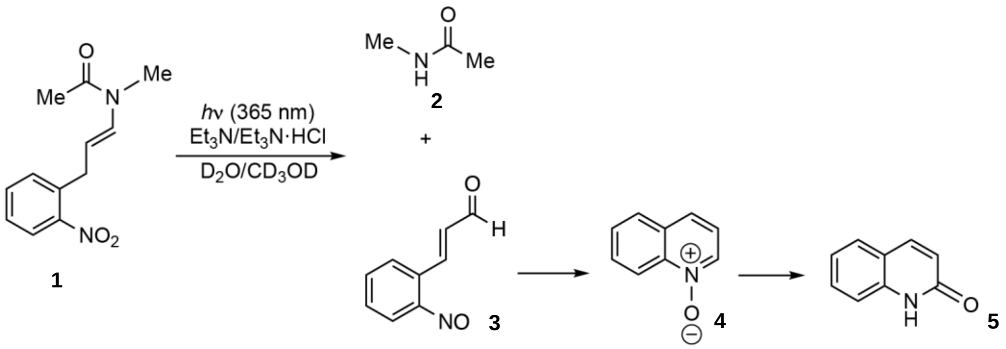

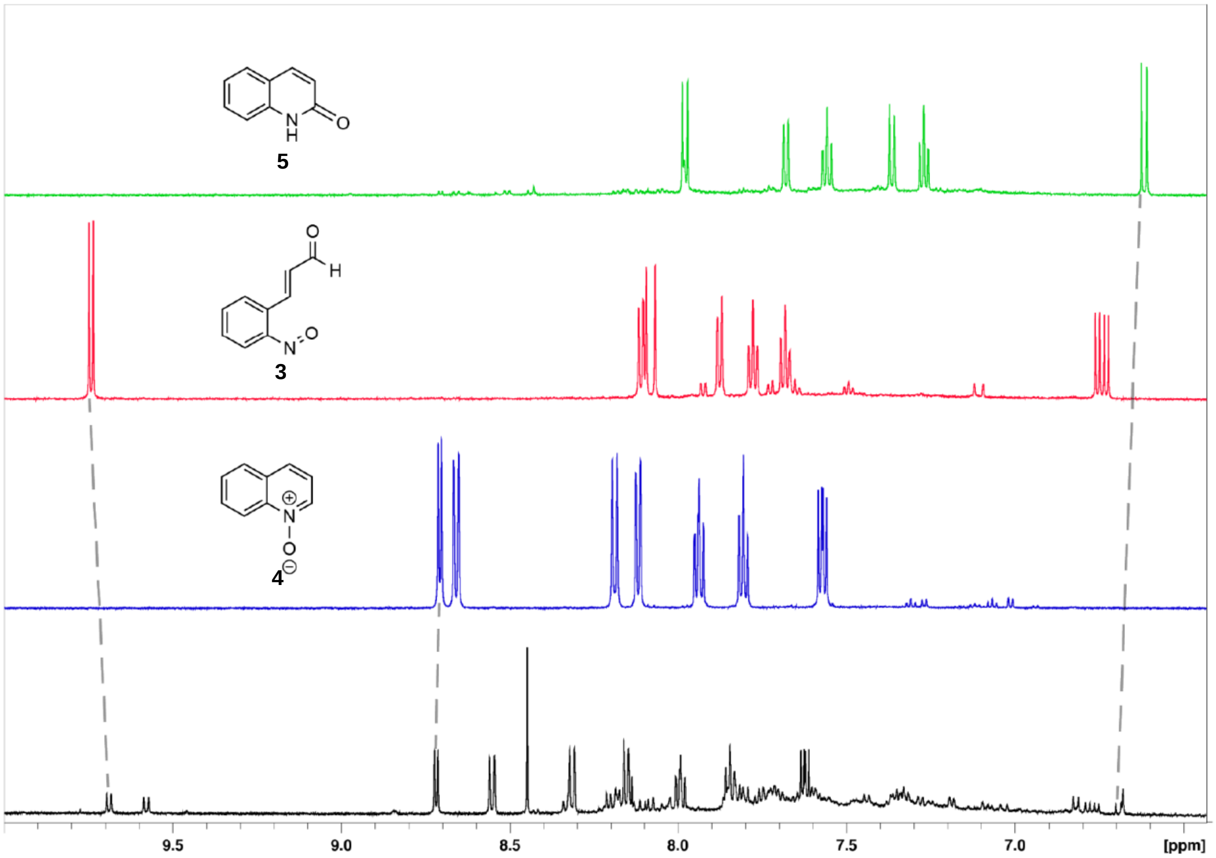

Figure S2. ^{1}H NMR spectra of crude reaction mixture (bottom) and isolated byproducts from photocleavage of compound **1**. The grey dashed lines match the peaks of the purified byproducts to their counterparts in the crude.

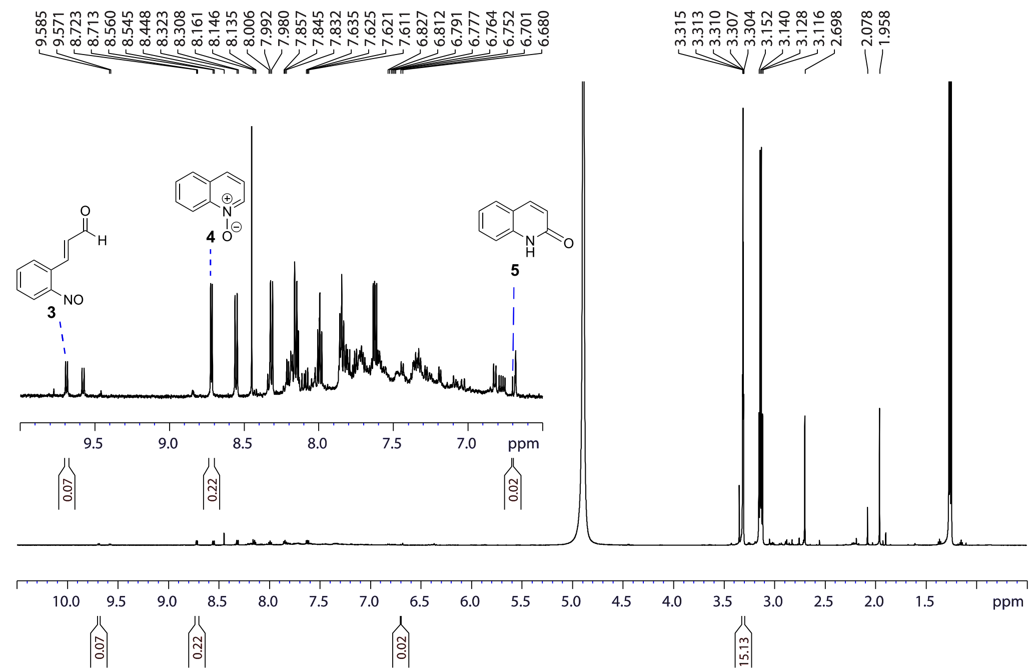

Figure S3. ^{1}H NMR spectrum of crude photocleavage of compound **1** under basic conditions at 15 min. The CD_3OD residual peaks at 3.31 ppm in both 0-min and 15-min spectra were integrated to 15.13. The NMR yield of **3**, **4** and **5** were calculated to be 7%, 22% and 4% (only one peak in the doublet of **5** was integrated), respectively.

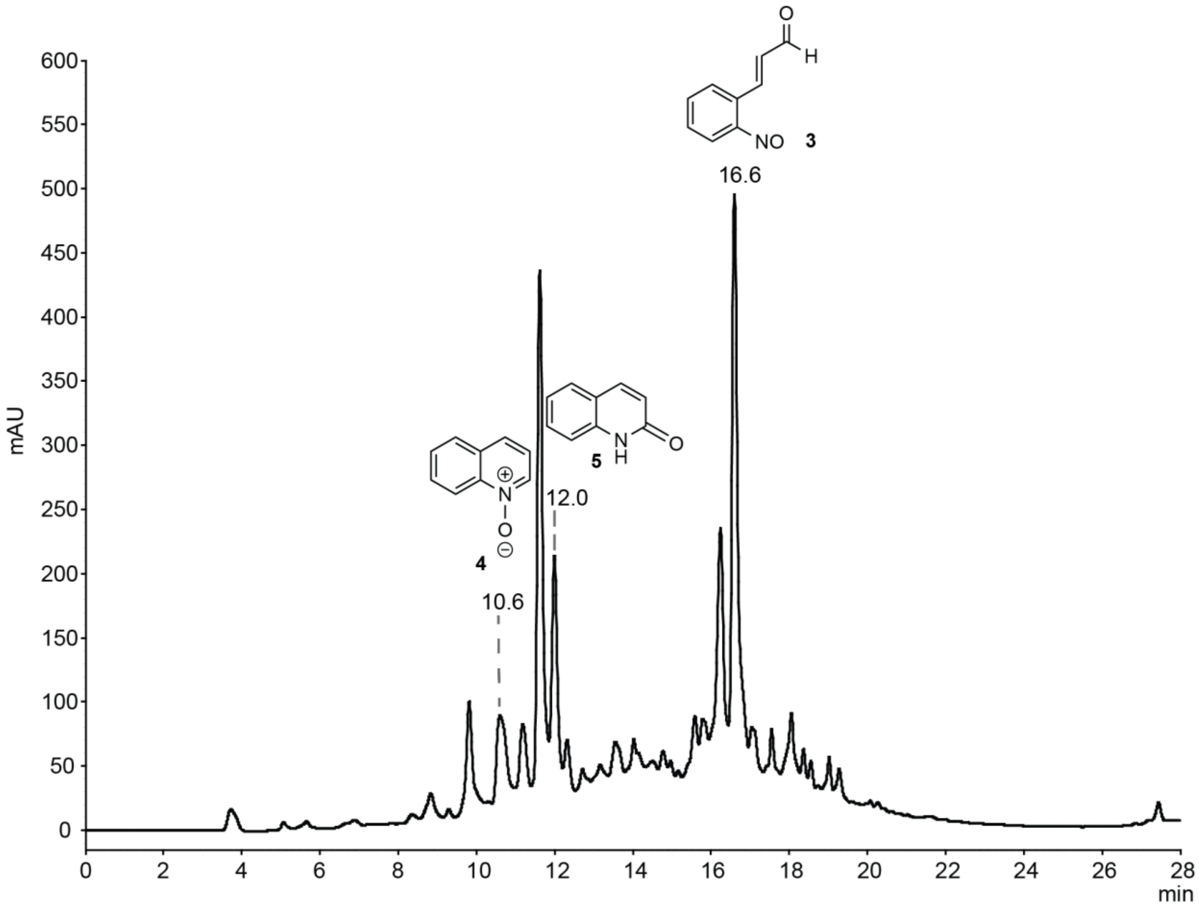

Figure S4. RP-HPLC trace of the crude photocleavage of compound **6**. Ramp: 15-90% MeCN 22 mins. Absorbance: 280 nm.

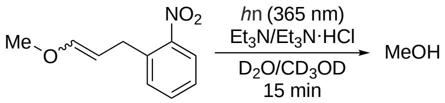

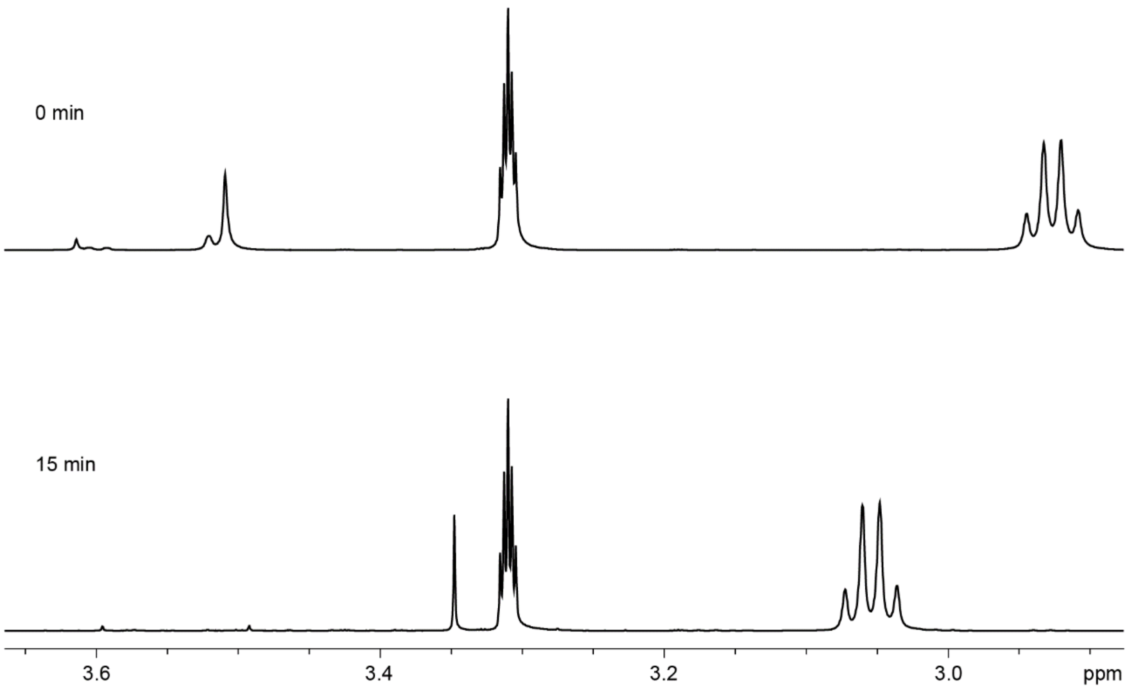

Figure S5. ^{1}H NMR spectrum compound **6** in D_2O/CD_3OD, triethylamine buffer at 0 min and 15 min irradiation (3.65 to 2.9 ppm). Peak at 3.348 ppm corresponds to one of the photocleavage products—MeOH.

For figure 3

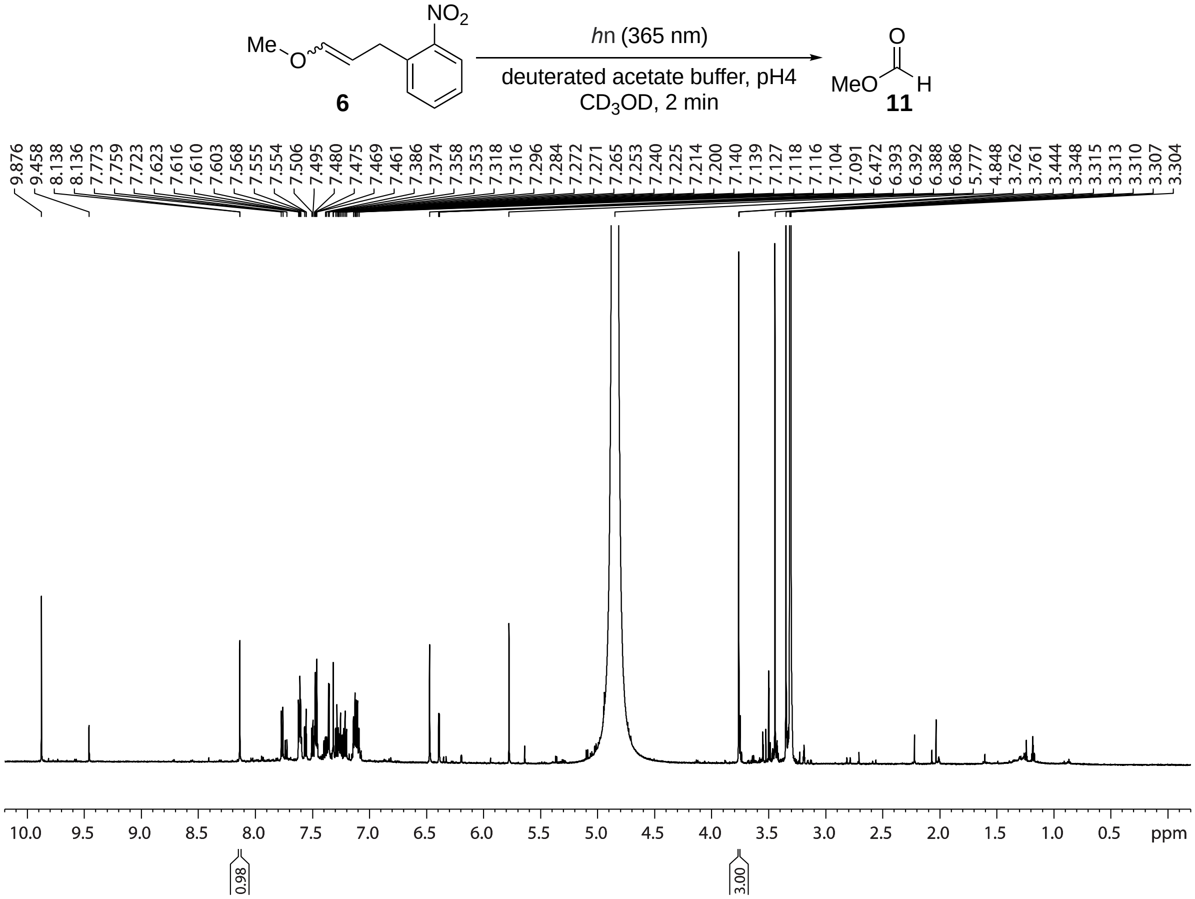

Figure S6. Compound **6** in CD_3OD and deuterated acetate buffer pH 4 at 2 min irradiation. Peak at 3.76 ppm and 8.14 ppm correspond to methyl formate **11**.

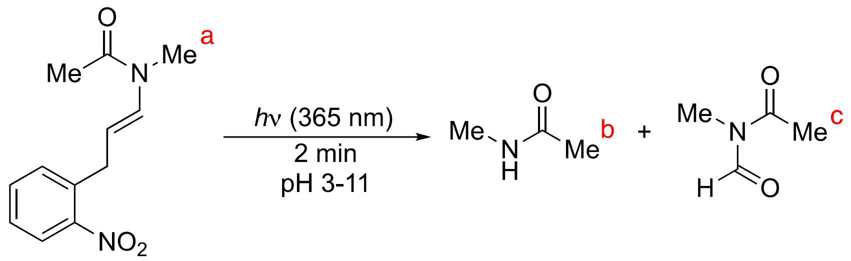

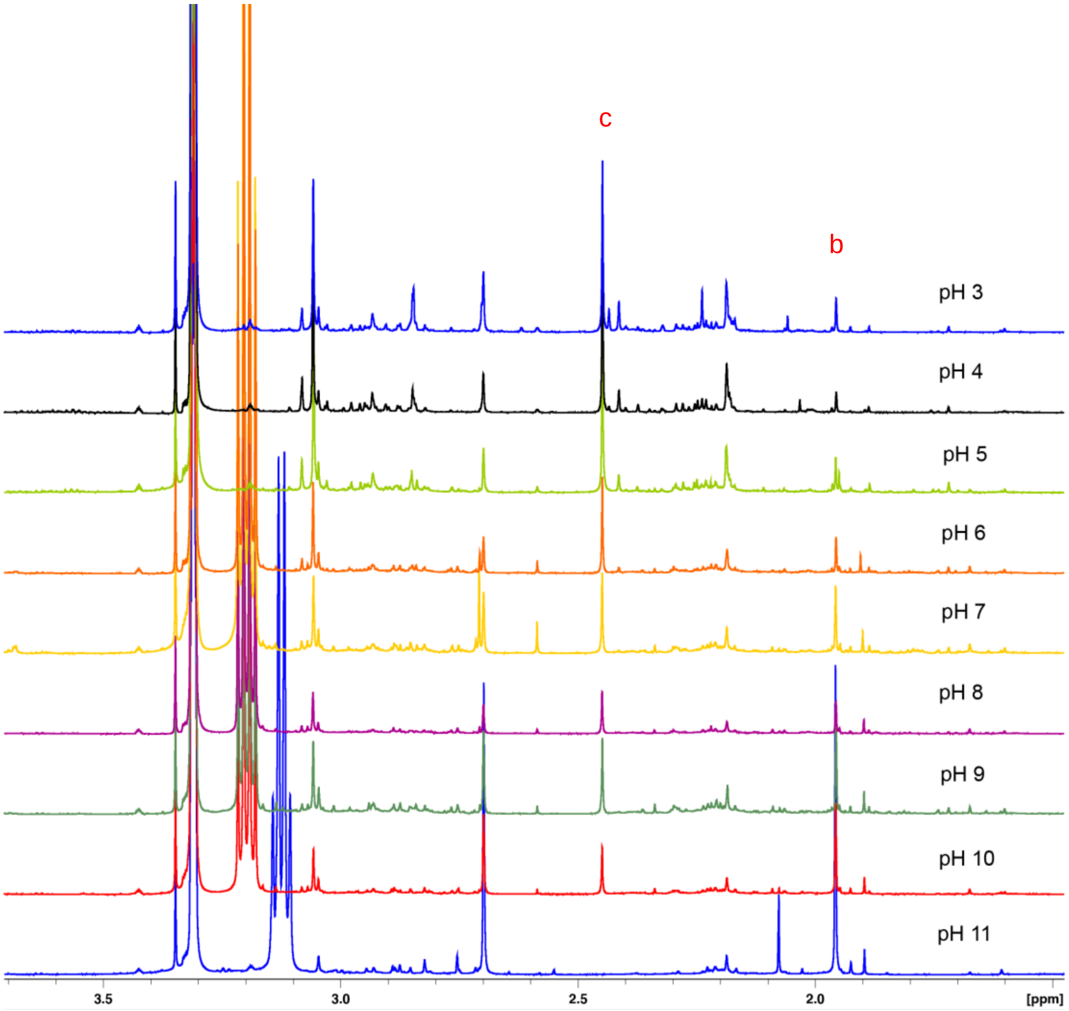

Figure S7. ^{1}H NMR study for dependance of yield of *N*-formyl-*N*-methyl acetamide **10** on pH of reaction buffer. Peak b was integrated to 1 in each spectrum and the integration for peak c was noted. The small methanol peak was present at 0 min irradiation, so it was not a product of the reaction.

PH	[N-methyl acetamide]	[N-formyl-N-methyl acetamide]	% formyl
**3**	1	4.88	83
**4**	1	8.59	90
**5**	1	6.5	87
**6**	1	2.76	73
**7**	1	1.34	57
**8**	1	1.55	61
**9**	1	0.88	47
**10**	1	0.56	36
**11**	1	0	0

Table S1. Yield of *N*-formyl-*N*-methyl acetamide **10** as a function of pH of buffer. The percentage of formyl was calculated from the ratio of [*N*-methyl acetamide] : [*N*-formyl-*N*-methyl acetamide].

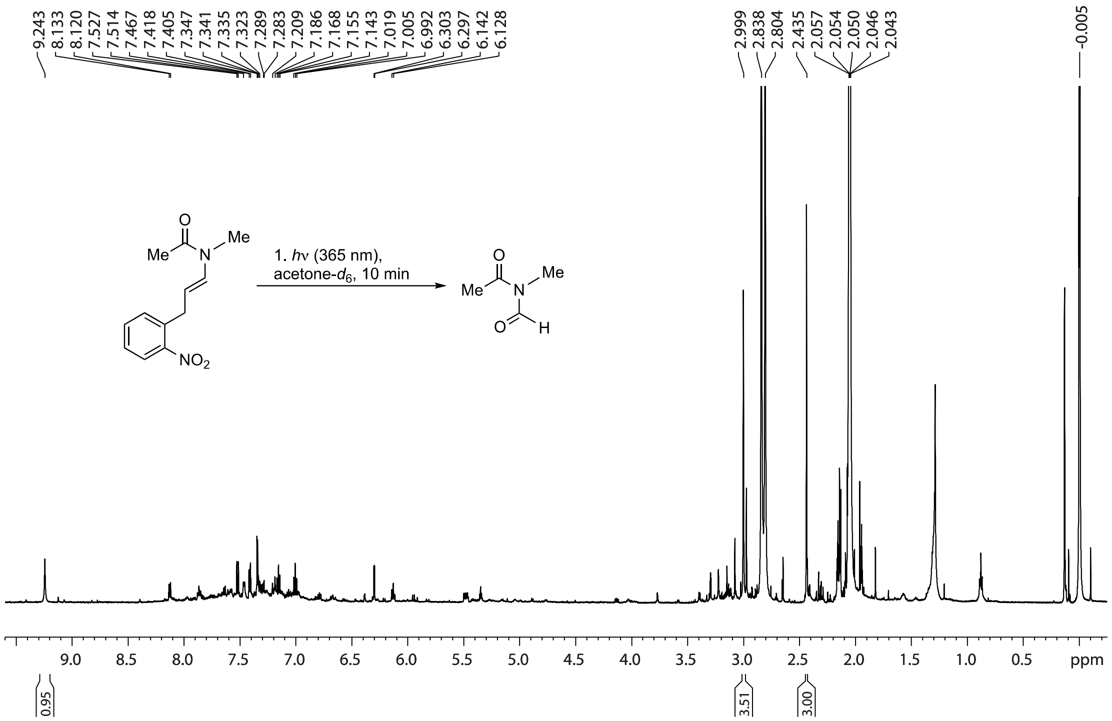

Figure S8. Compound **1** in acetone-d_6 at 10 min irradiation. Peak at 2.435 ppm and 9.243 ppm correspond to one of the photocleavage products— *N*-formyl-*N*-methyl acetamide **10**.

For figure 4

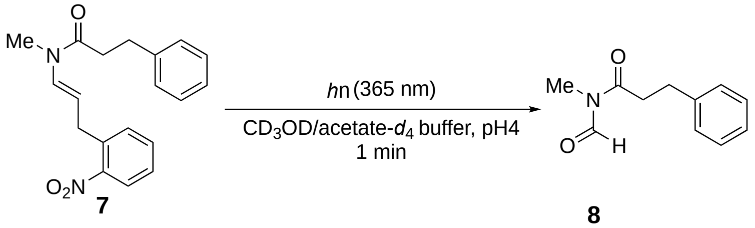

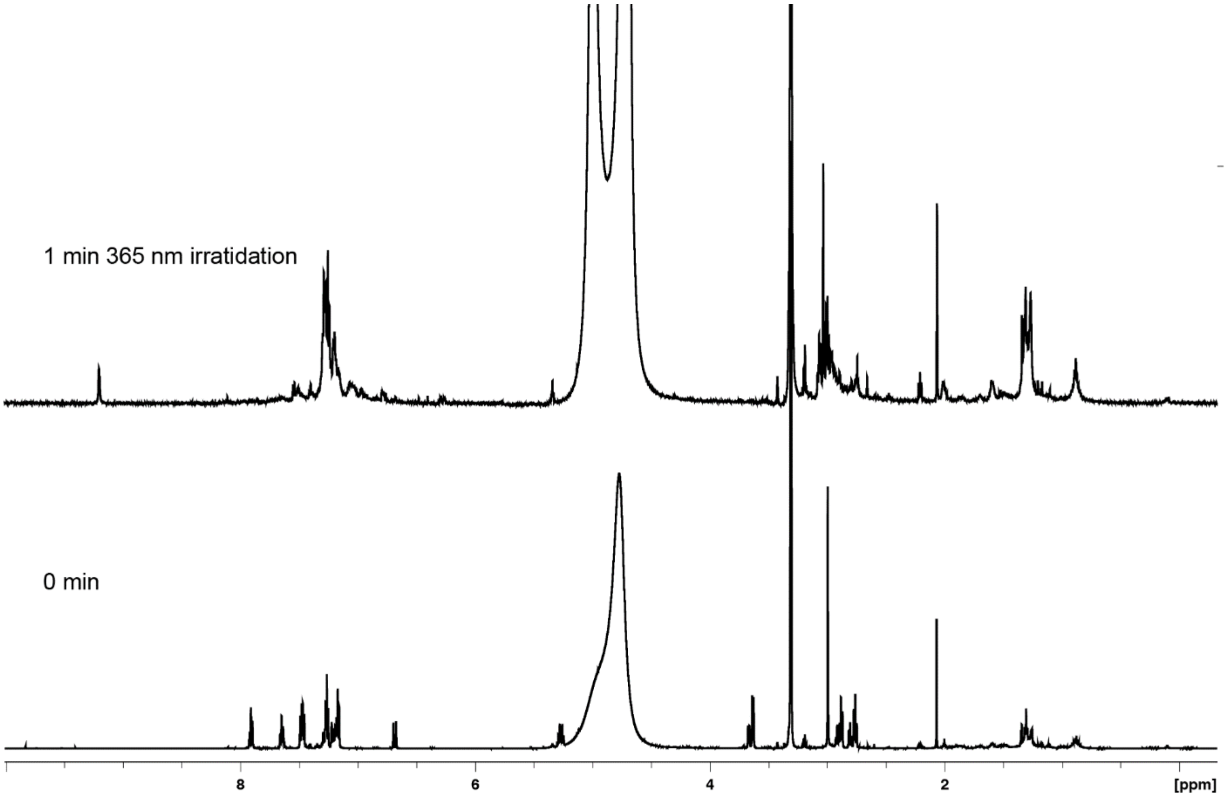

Figure S9. ^{1}H NMR spectra of compound 7 at 0 min (bottom), and after 1 min of irradiation at 365 nm (top).

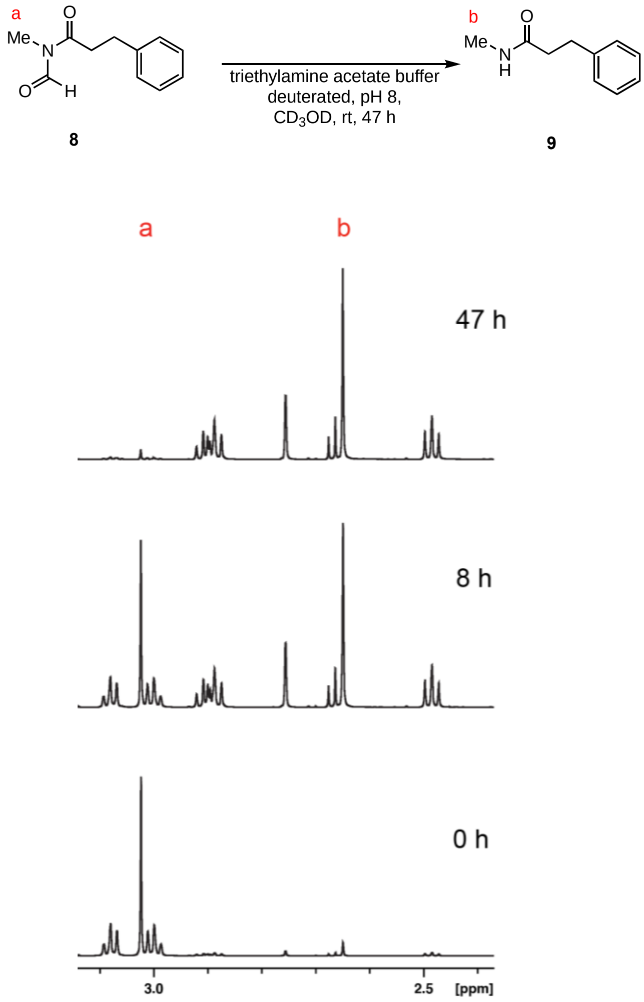

Figure S10. Comparison of ^{1}H NMR spectra of degradation of **8** at 0 h, 8 h and 47 h. Major degradation product was compound **9**.

Obtaining the first-order rate constant of degradation of compound 8

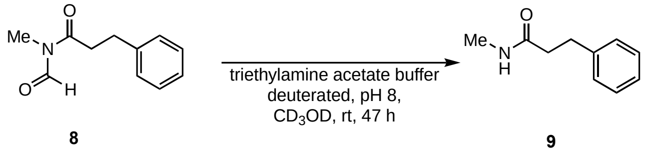

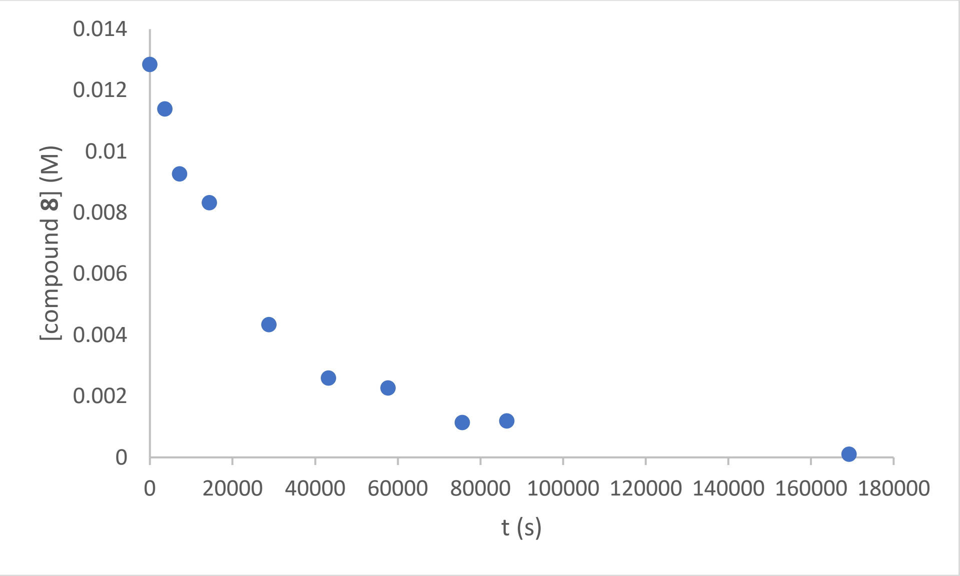

Figure S11. Plot of decrease of compound **8** over time.

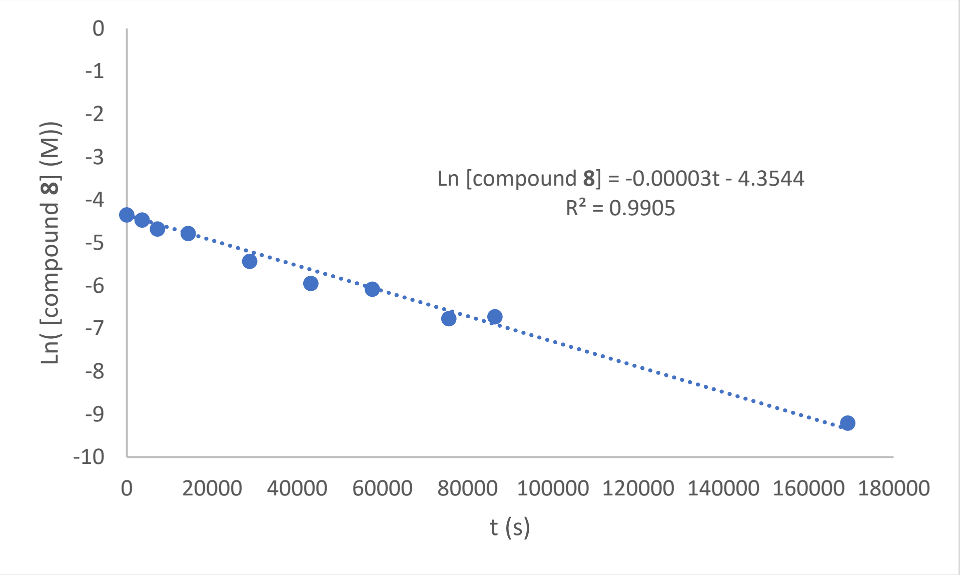

Figure S12. Plot of Ln [compound **8**] vs. time. The rate constant is 0.00003 s^{-1}. The half-life ($t_{1/2}$) was calculated to be 23100 s or 6.42 h.

Compound Characterization

HPLC of synthesized compounds

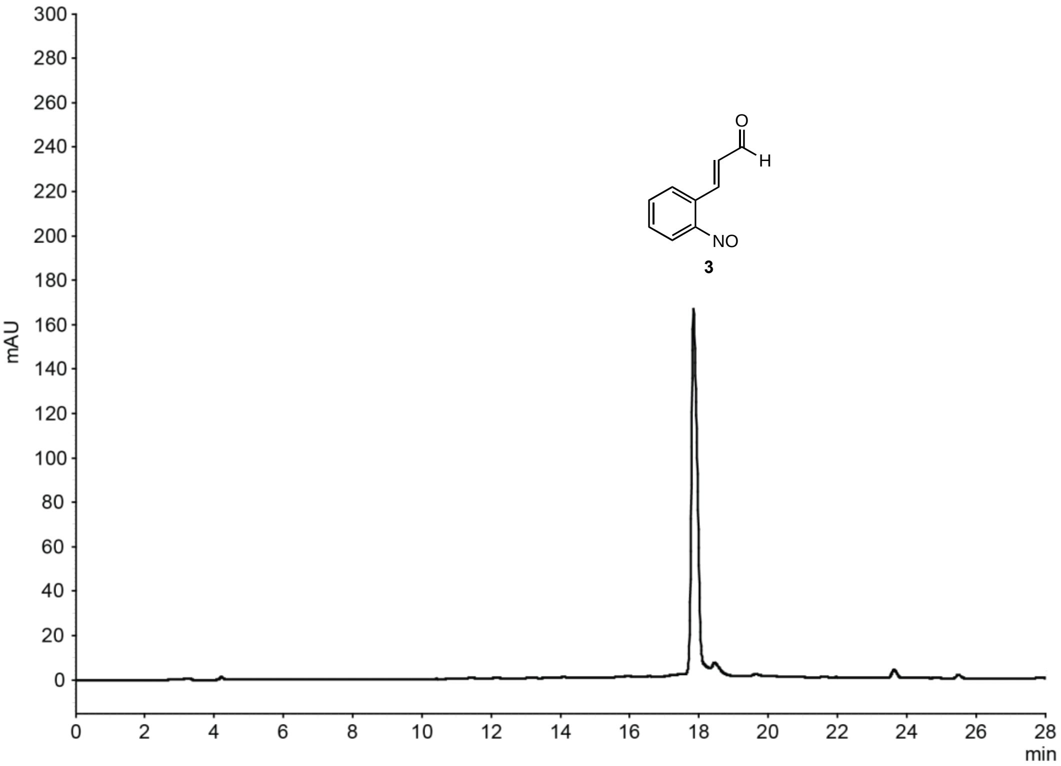

Figure S13. HPLC chromatogram of purified compound **3**. Ramp: 15-90% MeCN 22 mins. Absorbance: 280 nm.

ESI-MS of synthesized compounds

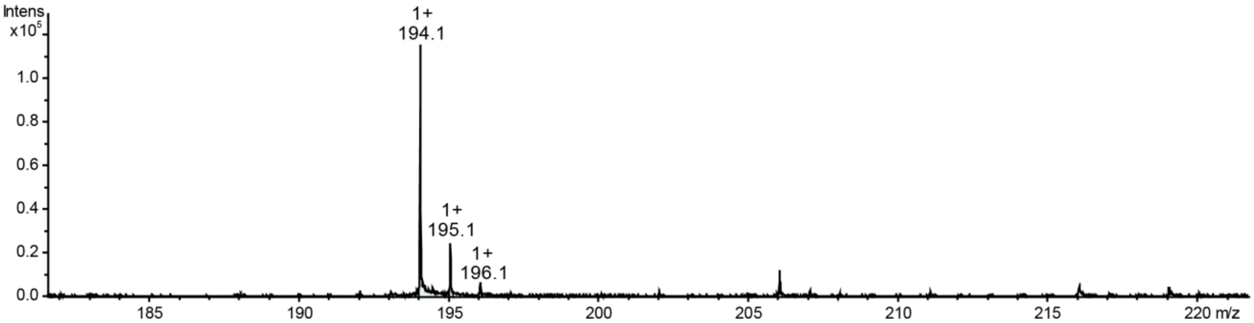

Figure S14. 1-(3-methoxyallyl)-2-nitrobenzene (**6**) (positive mode). m/z 194.1 corresponds to $[M+H]^{+}$.

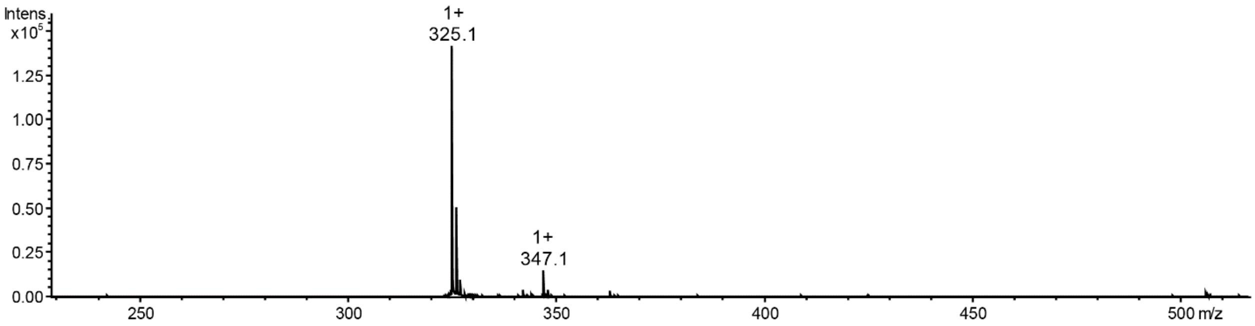

Figure S15. (*E*)-*N*-methyl-*N*-(3-(2-nitrophenyl)prop-1-en-1-yl)-3-phenylpropanamide (**7**) (positive mode). m/z 325.1 corresponds to $[M+H]^+$, and m/z 347.1 corresponds to $[M+Na]^+$.

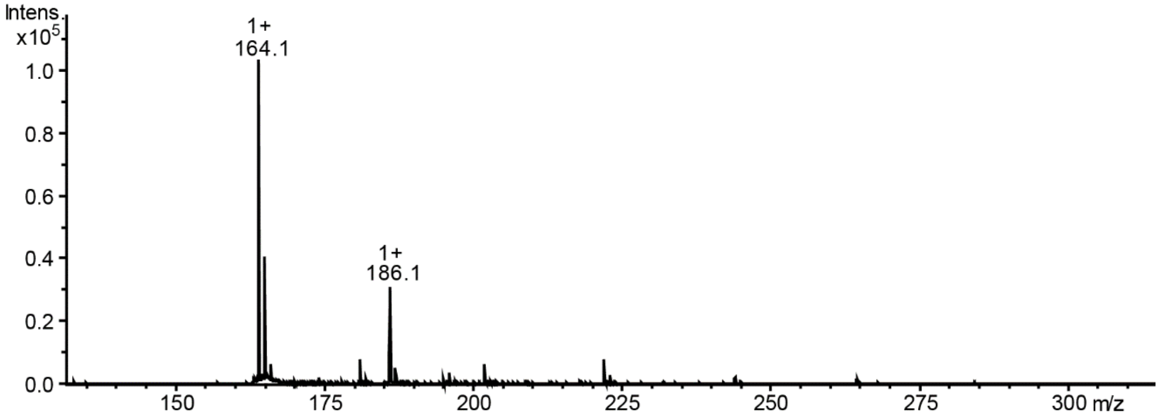

Figure S16. *N*-Methyl-3-phenylpropionamide (**9**) (positive mode). m/z 164.1 corresponds to $[M+H]^+$.

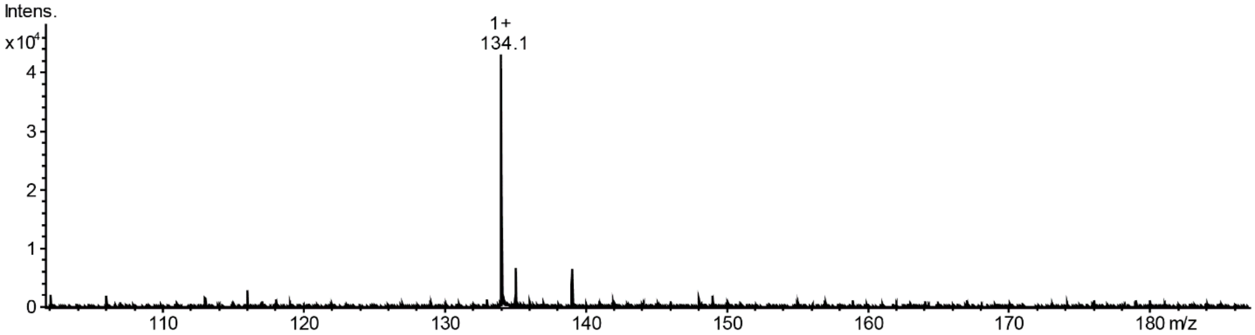

Figure S17. 1H-indol-1-yl acetate (**12**) (positive mode). m/z 134.1 corresponds to $[M-Ac+2H]^+$.

^{1}H and ^{13}C NMR spectra of purified compounds

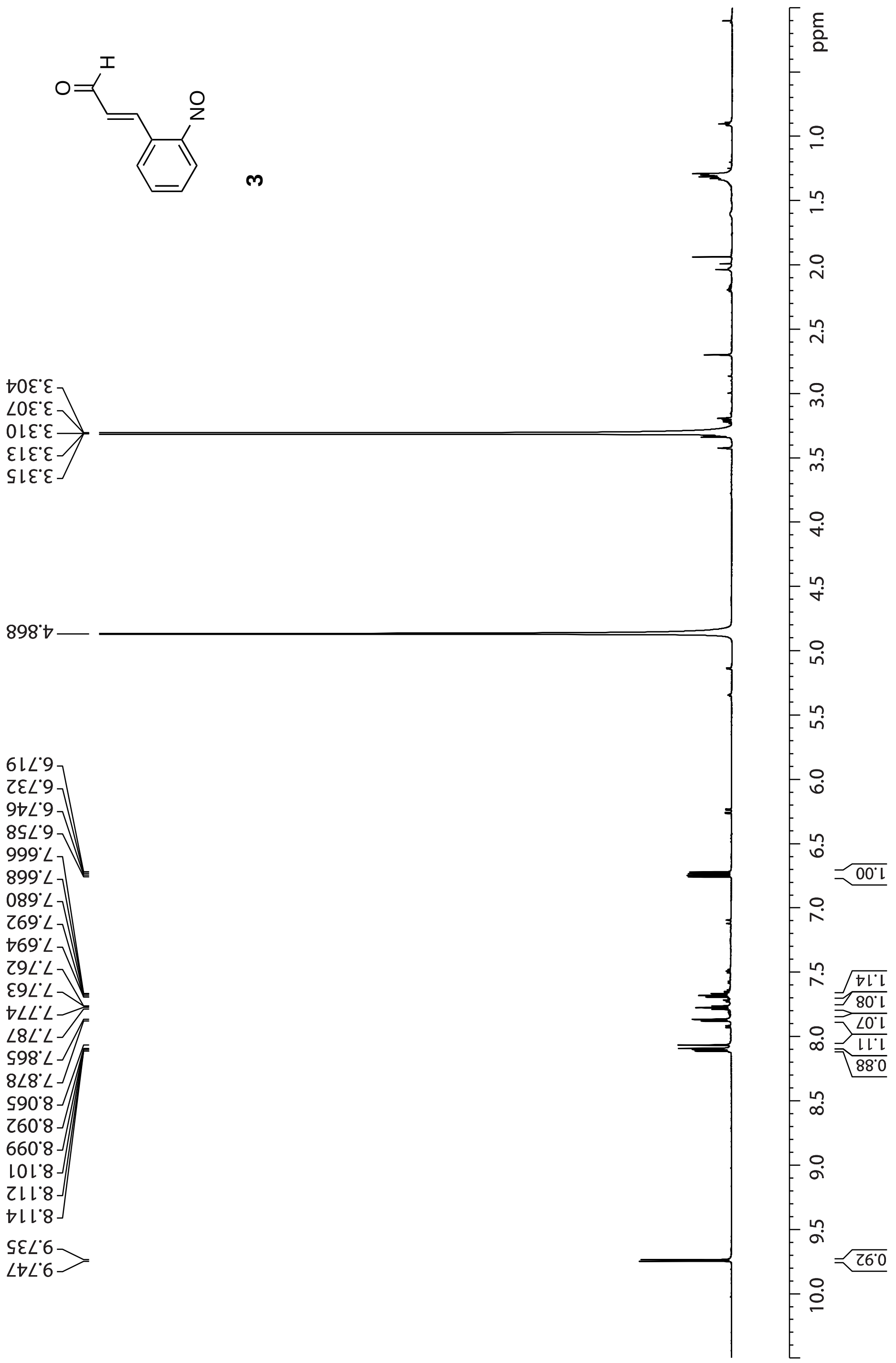

Figure S18. ^{1}H NMR spectrum of (*E*)-3-(2-nitrosophenyl)acrylaldehyde **3** in CD_3OD

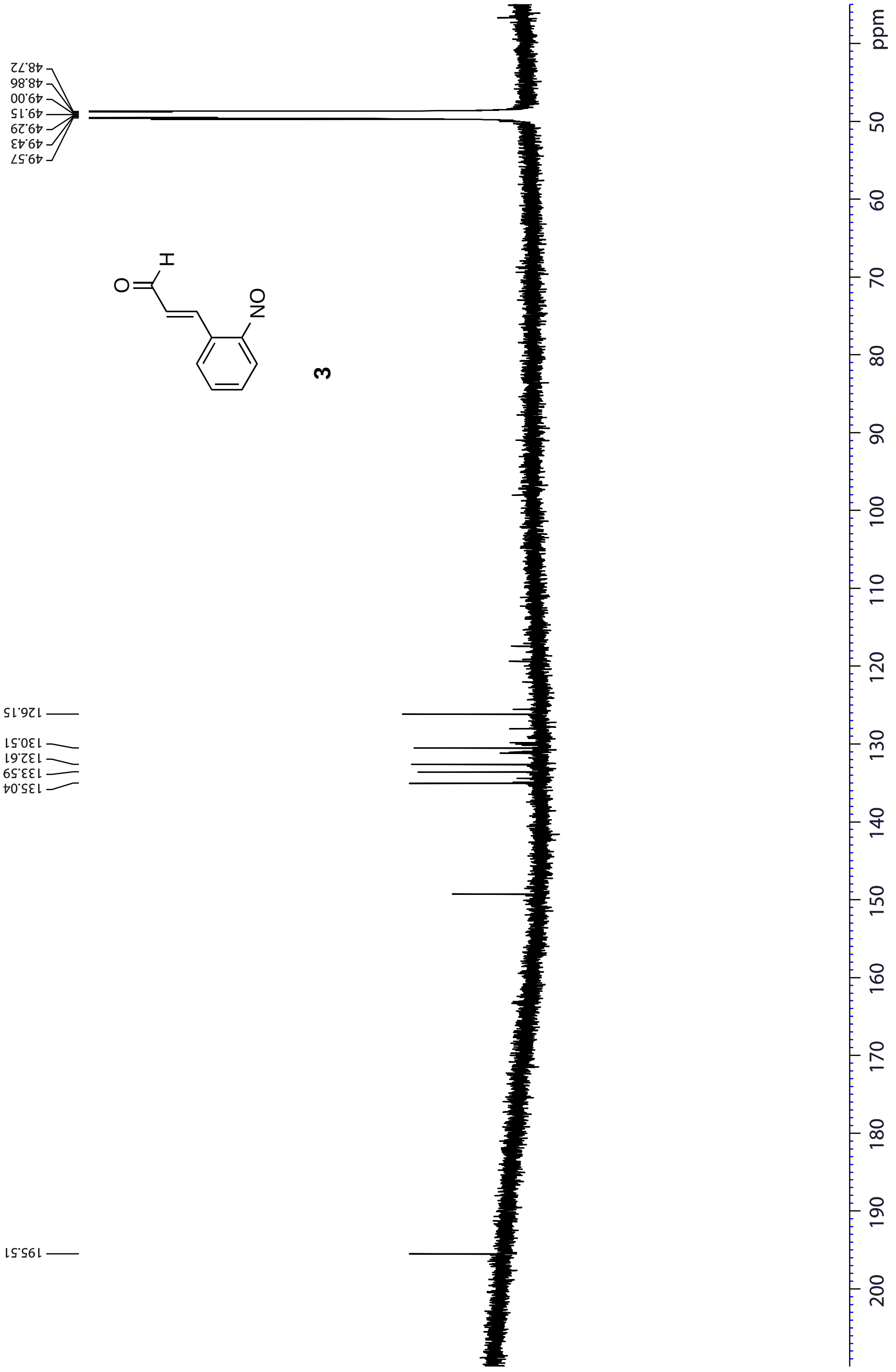

Figure S19. ^{13}C NMR spectrum of (*E*)-3-(2-nitrosophenyl)acrylaldehyde **3** in CD_3OD

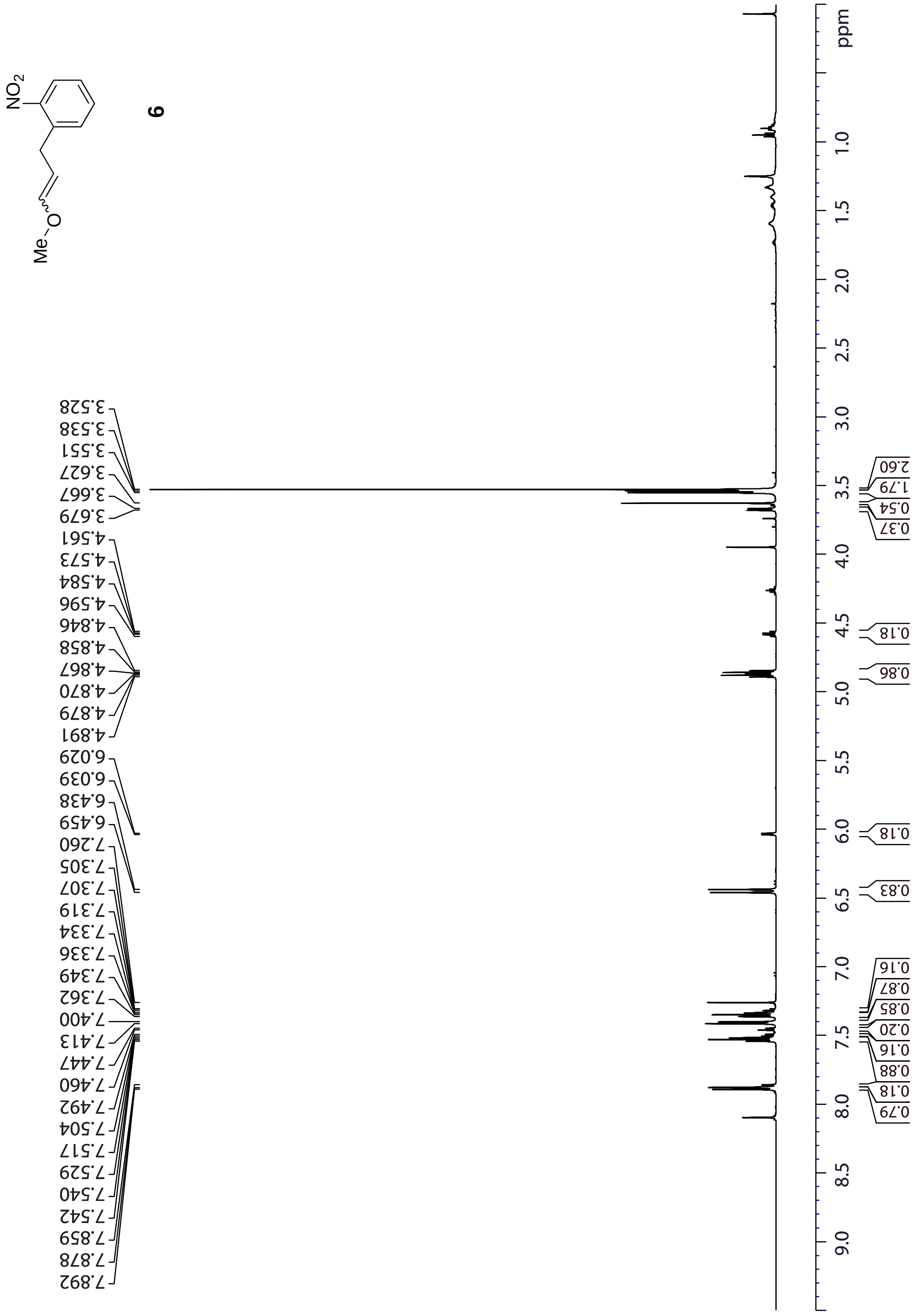

Figure S20. ^{1}H NMR spectrum of 1-(3-methoxyallyl)-2-nitrobenzene **6** in $CDCl_3$

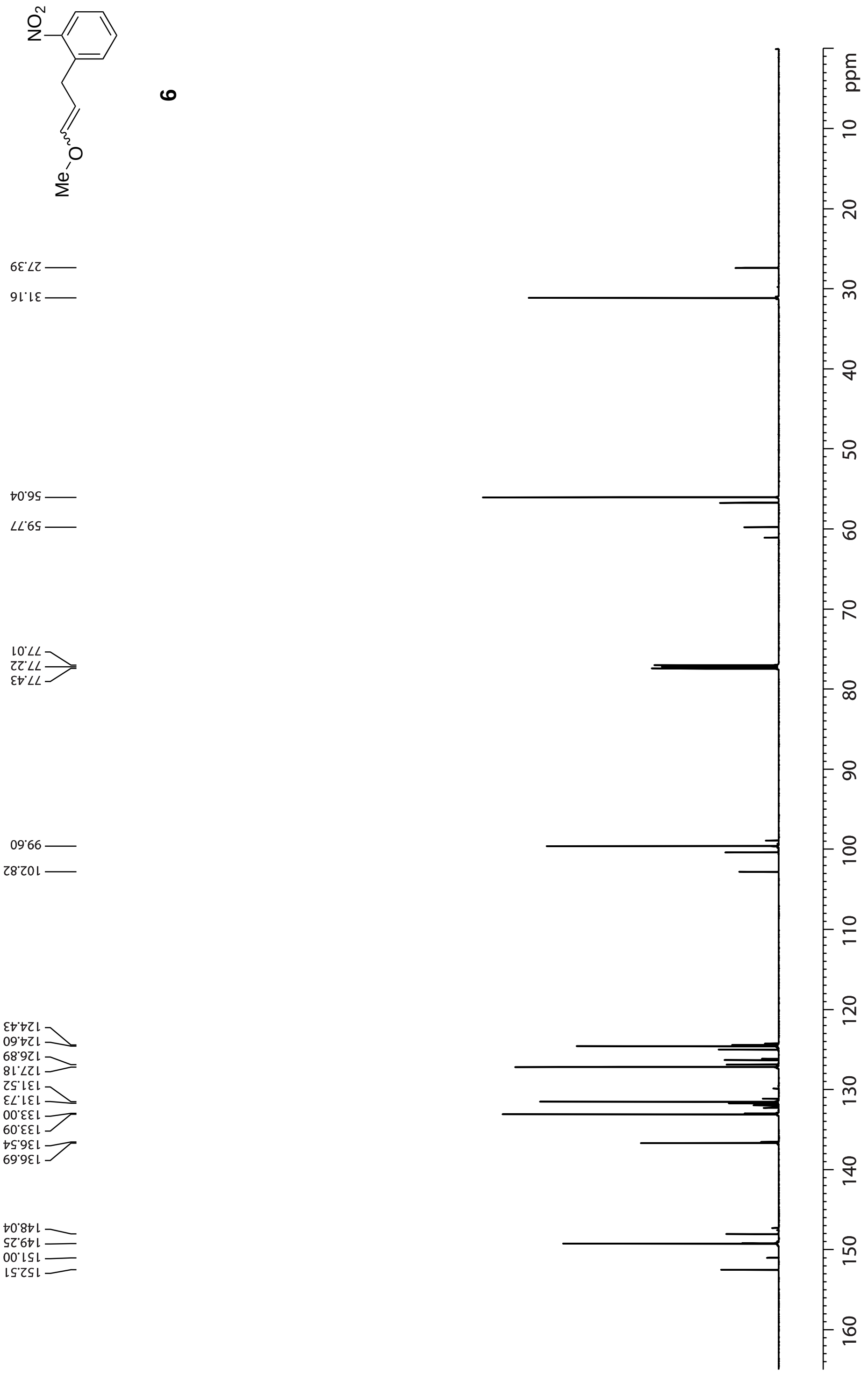

Figure S21. ^{13}C NMR spectrum of 1-(3-methoxyallyl)-2-nitrobenzene **6** in $CDCl_3$

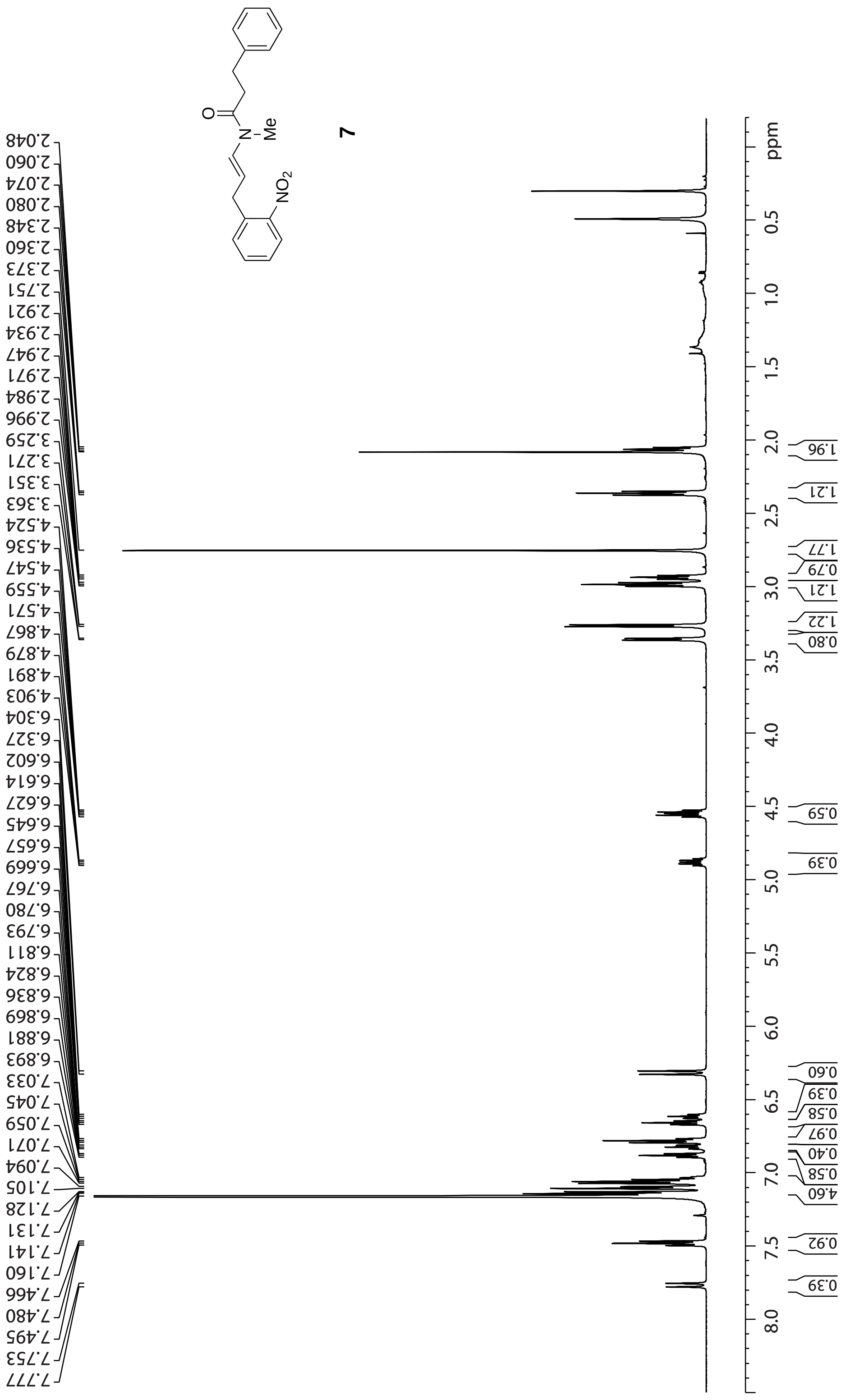

Figure S22. ^{1}H NMR spectrum of (*E*)-*N*-methyl-*N*-(3-(2-nitrophenyl)prop-1-en-1-yl)-3-phenylpropanamide **7** in C_6D_6

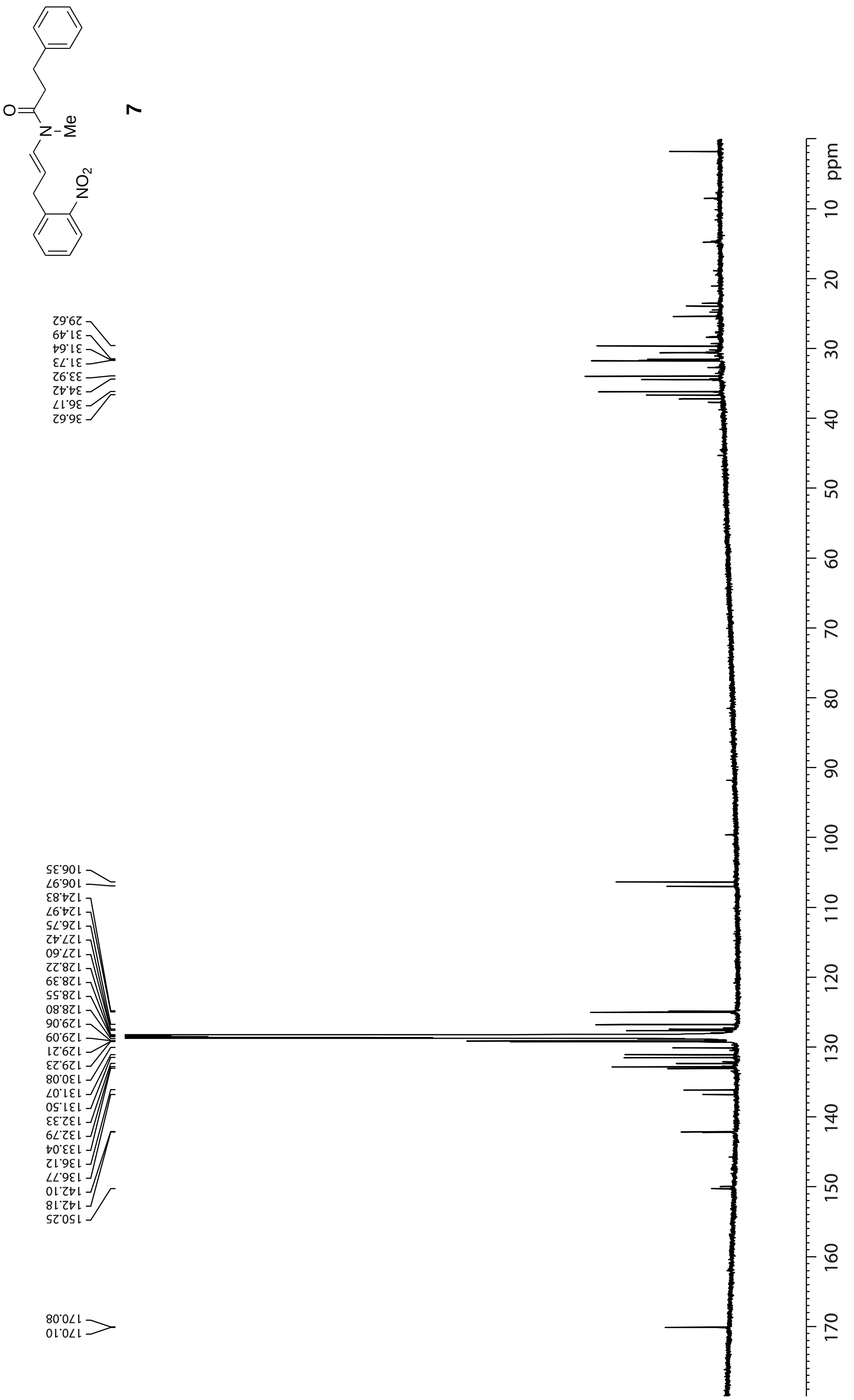

Figure S23. ^{13}C NMR spectrum of (*E*)-*N*-methyl-N-(3-(2-nitrophenyl)prop-1-en-1-yl)-3-phenylpropanamide **7** in C_6D_6

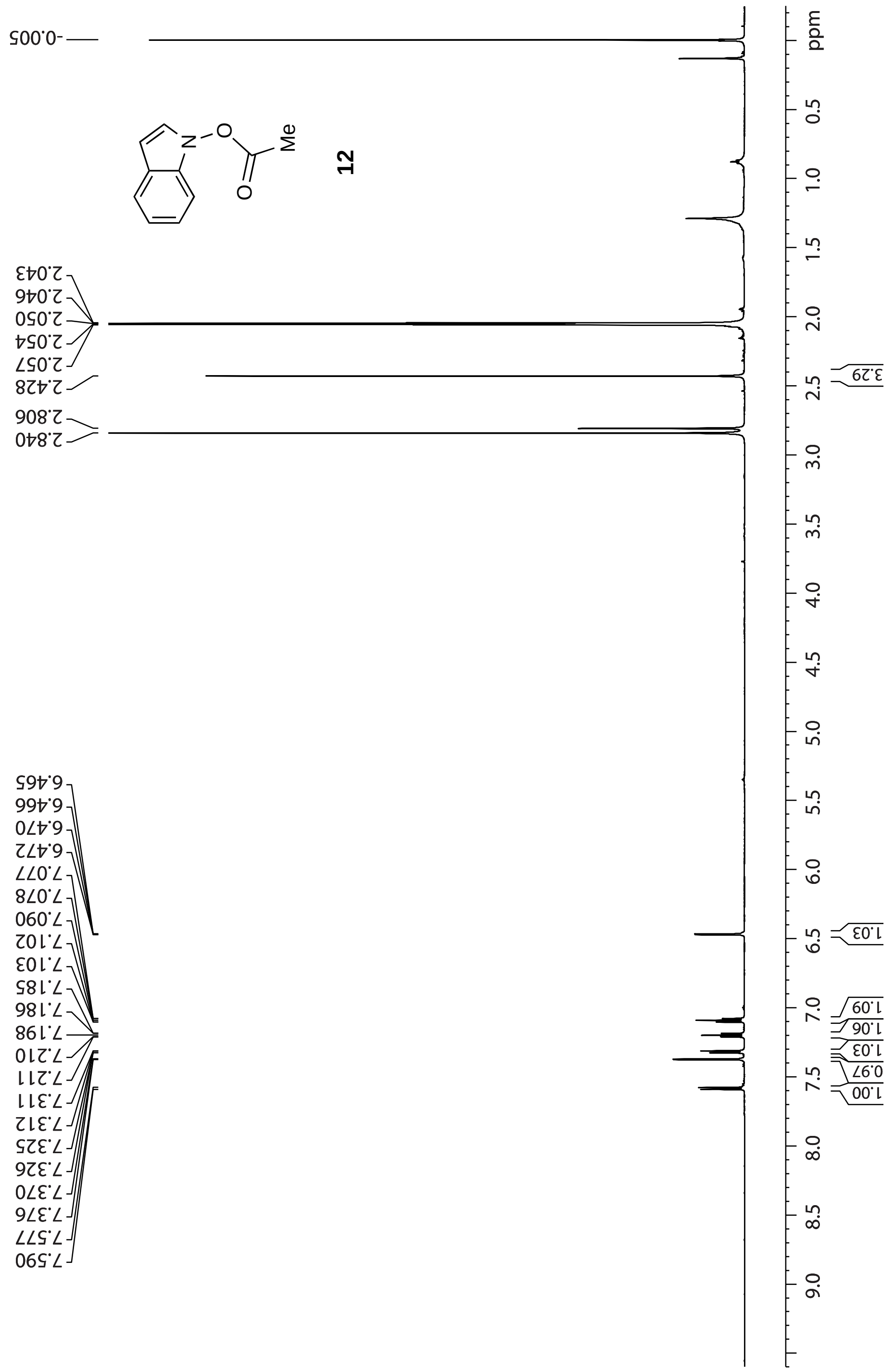

Figure S24. ^{1}H NMR spectrum of 1H-indol-1-yl acetate **(12)** in acetone-d_6.

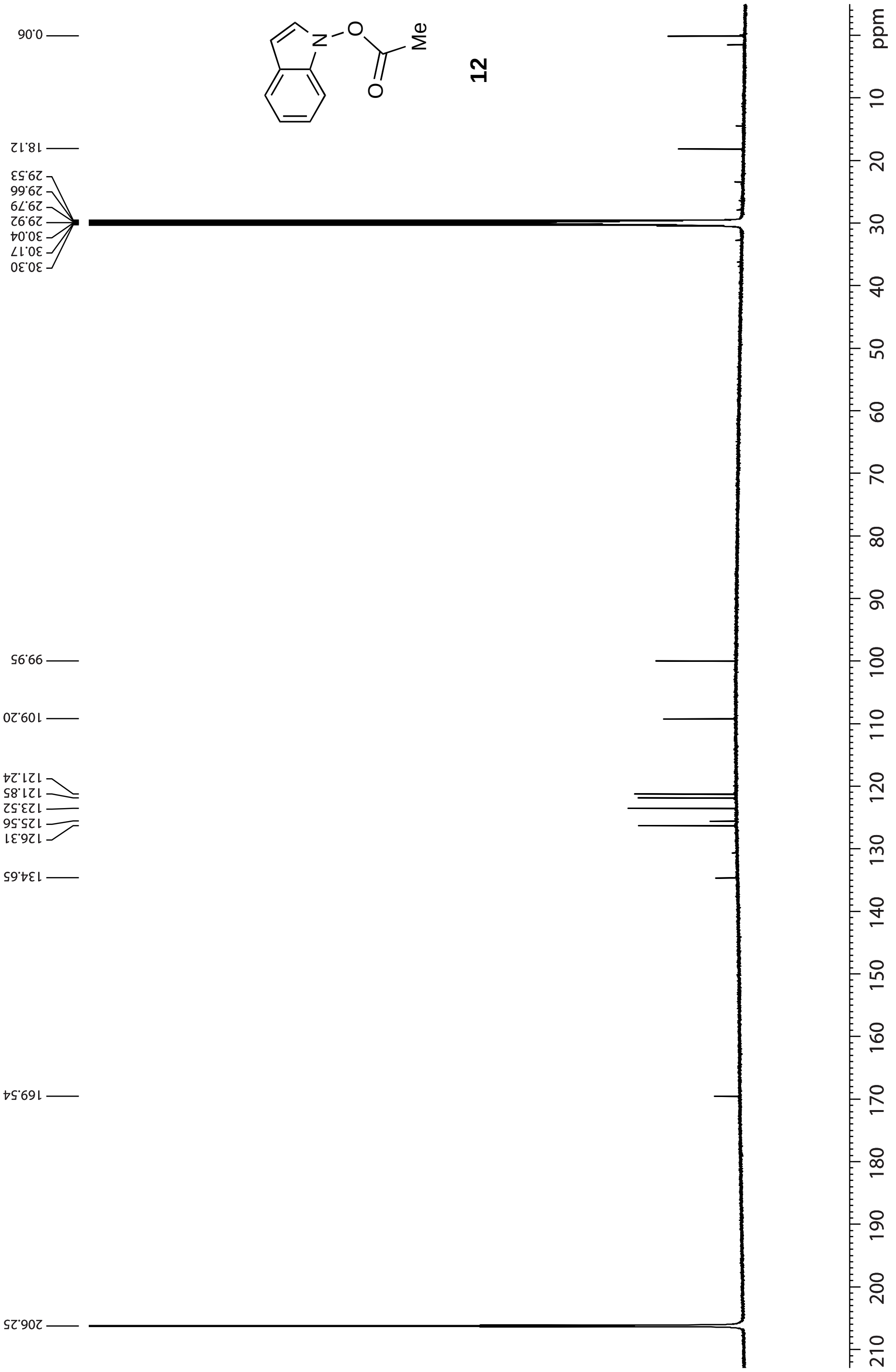

Figure S25. ^{13}C NMR spectrum of 1H-indol-1-yl acetate (**12**) in acetone-d_6.

References

(1) Mangubat-Medina, A. E.; Martin, S. C.; Hanaya, K.; Ball, Z. T. A Vinylogous Photocleavage Strategy Allows Direct Photocaging of Backbone Amide Structure. *J. Am. Chem. Soc.* **2018**. https://doi.org/10.1021/jacs.8b04893.

(2) Ramos, J. A. F.; Araújo, C. S.; Nagem, T. J.; Taylor, J. G. Synthesis of Indolines via a SmI2 Promoted Domino Nitro Reduction–Intramolecular Aza-Michael Reaction. *J. Heterocycl. Chem.* **2015**, *52* (1), 54–58. https://doi.org/10.1002/jhet.1982.

(3) Hall, L. R.; Iwamoto, R. T.; Hanzlik, R. P. Electrochemical Models for Cytochrome P-450. N-Demethylation of Tertiary Amides by Anodic Oxidation. *J. Org. Chem.* **1989**, *54* (10), 2446–2451. https://doi.org/10.1021/jo00271a040.

(4) Molina, P.; Alajarín, M.; Sánchez-Andrada, P.; Carrió, J. S.; Martínez-Ripoll, M.; Anderson, J. E.; Jimeno, M. L.; Elguero, J. New Models for the Study of the Racemization Mechanism of Carbodiimides. Synthesis and Structure (X-Ray Crystallography and 1H NMR) of Cyclic Carbodiimides. *J. Org. Chem.* **1996**, *61* (13), 4289–4299. https://doi.org/10.1021/jo951789c.

(5) Rodrigues, T.; Reker, D.; Kunze, J.; Schneider, P.; Schneider, G. Revealing the Macromolecular Targets of Fragment-Like Natural Products. *Angew. Chem. Int. Ed.* **2015**, *54* (36), 10516–10520. https://doi.org/10.1002/anie.201504241.

(6) Li, Y.; Wong, L. L. Multi-Functional Oxidase Activity of CYP102A1 (P450BM3) in the Oxidation of Quinolines and Tetrahydroquinolines. *Angew. Chem. Int. Ed.* **2019**, *58* (28), 9551–9555. https://doi.org/10.1002/anie.201904157.

(7) Acheson, R. M.; Hunt, P. G.; Littlewood, D. M.; Murrer, B. A.; Rosenberg, H. E. The Synthesis, Reactions, and Spectra of 1-Acetoxy-, 1-Hydroxy-, and 1-Methoxy-Indoles. *J. Chem. Soc. Perkin 1* **1978**, No. 10, 1117–1125. https://doi.org/10.1039/P19780001117.

(8) Fulmer, G. R.; Miller, A. J. M.; Sherden, N. H.; Gottlieb, H. E.; Nudelman, A.; Stoltz, B. M.; Bercaw, J. E.; Goldberg, K. I. NMR Chemical Shifts of Trace Impurities: Common Laboratory Solvents, Organics, and Gases in Deuterated Solvents Relevant to the Organometallic Chemist. *Organometallics* **2010**, *29* (9), 2176–2179. https://doi.org/10.1021/om100106e.

(9) Babij, N. R.; McCusker, E. O.; Whiteker, G. T.; Canturk, B.; Choy, N.; Creemer, L. C.; Amicis, C. V. D.; Hewlett, N. M.; Johnson, P. L.; Knobelsdorf, J. A.; Li, F.; Lorsbach, B. A.; Nugent, B. M.; Ryan, S. J.; Smith, M. R.; Yang, Q. NMR Chemical Shifts of Trace Impurities: Industrially Preferred Solvents Used in Process and Green Chemistry. *Org. Process Res. Dev.* **2016**, *20* (3), 661–667. https://doi.org/10.1021/acs.oprd.5b00417.

(10) Rong, Z.-Q.; Zhang, Y.; Chua, R. H. B.; Pan, H.-J.; Zhao, Y. Dynamic Kinetic Asymmetric Amination of Alcohols: From A Mixture of Four Isomers to Diastereo- and Enantiopure α-Branched Amines. *J. Am. Chem. Soc.* **2015**, *137* (15), 4944–4947. https://doi.org/10.1021/jacs.5b02212.

(11) Hanada, S.; Ishida, T.; Motoyama, Y.; Nagashima, H. The Ruthenium-Catalyzed Reduction and Reductive N-Alkylation of Secondary Amides with Hydrosilanes: Practical Synthesis of Secondary and Tertiary Amines by Judicious Choice of Hydrosilanes. *J. Org. Chem.* **2007**, *72* (20), 7551–7559. https://doi.org/10.1021/jo070591c.
